# Deep learning for optoacoustic imaging of reversibly switchable proteins: training and performance testing using simulated multispectral optoacoustic tomography images

**DOI:** 10.1117/1.JBO.31.8.086002

**Published:** 2026-08-10

**Authors:** William Vale, Jeffrey Bamber, Hasan Koruk, Gustavo Carneiro, Lucia Florescu

**Affiliations:** a University of Surrey, Centre for Vision, Speech & Signal Processing, Faculty of Engineering and Physical Sciences, Guildford, United Kingdom; b The Institute of Cancer Research, Division of Radiotherapy and Imaging, Joint Department of Physics, London, United Kingdom; c National Physical Laboratory, Department of Medical, Marine and Nuclear, Ultrasound and Underwater Acoustics Group, London, United Kingdom

**Keywords:** optoacoustic imaging, reversibly switchable optoacoustic proteins, chimeric antigen receptor T-cells, deep learning, Monte Carlo eXtreme, k-wave

## Abstract

**Significance:**

Reversibly switchable optoacoustic proteins (rsOAPs) are a promising candidate for sensitive and quantitative optoacoustic (OA) imaging of genetically modified cell populations, such as chimeric antigen receptor (CAR) T-cells used in cancer immunotherapy. Although detection of rsOAPs has been demonstrated using classical machine learning approaches, there is still a need for higher detection sensitivity and more accurate quantification.

**Aim:**

We aim to develop deep learning approaches for improving the sensitivity of the detection and accuracy of quantification of rsOAPs with OA imaging and a 3D simulation framework to create synthetic datasets for machine learning experiments.

**Approach:**

We developed a forward model to generate labeled synthetic OA images of rsOAPs that takes into account light transport, acoustic wave propagation, and light-driven transitions between two different forms of the proteins. We used the synthetic images to train and evaluate machine learning models, including two convolutional neural networks and a transformer neural network, on the binary semantic segmentation and pixel-level prediction (regression) of the spatial distribution of the rsOAPs.

**Results:**

With this dataset, fine-tuned convolutional and transformer neural networks substantially outperformed classical machine learning approaches in the binary semantic segmentation of rsOAPs within the inhomogeneities (regions representing tumors), increasing the sensitivity from around 0.38 to 0.55 for noiseless data and from around 0.15 to 0.53 under a high level of stochastic noise (${\mathrm{SNR}}_{\mathrm{dB}}=8.8$) while maintaining a specificity of around 0.84, down to protein concentrations of order $10^{-8}\;M$.

**Conclusions:**

A new methodology for the generation of synthetic OA imaging data of rsOAPs is presented, and the feasibility of deep learning for the accurate semantic segmentation and quantification of rsOAPs in OA imaging is demonstrated.

## 1 Introduction

Chimeric antigen receptor (CAR) T-cell therapy is a form of cancer treatment in which a type of white blood cell, known as T-cells, is extracted from the patient, modified to attack cancer cells, and then inserted back into the patient. CAR T-cell therapy is an increasingly effective clinical treatment for hematological cancers but has only achieved limited success for solid tumors.^1^ To improve understanding of its efficacy for solid tumors, methods of quantifying the spatial distribution of CAR T-cells *in vivo* are needed.^2^ Optoacoustic (OA) imaging paired with genetically encoded reversibly switchable optoacoustic proteins (rsOAPs) is a promising candidate.^3^^–^^8^ OA imaging is a hybrid imaging modality that combines light and ultrasound, and is characterized by optical contrast, with higher penetration depths than purely optical techniques,^9^^–^^11^ rapid 3D imaging capability, and high spatial resolution when compared with magnetic resonance imaging^12^^,^^13^ and positron emission tomography.^14^

To be detectable in the body via medical imaging techniques, nonpigmented cells, including CAR T-cells, must be labeled with a contrast agent. Quantitative OA imaging of cells labeled with contrast agents, such as nanoparticles^13^^,^^15^ and dyes like indocyanine green,^16^ can be challenging due to the intermixing of strong background signals, image reconstruction artifacts, poor signal-to-noise ratio (SNR), and local fluence variations arising from wavelength-dependent attenuation (spectral coloring),^17^ which may confound spectral recognition (unmixing) algorithms. Furthermore, these contrast agents become diluted when the cells multiply as is the case for CAR T-cells. A promising approach to overcome these limitations involves transfecting the cells to incorporate a suitable reporter gene into their DNA, causing them to produce rsOAPs that absorb light at wavelengths in the near-infrared region, which may be altered by illumination at a switching wavelength. OA imaging is used to image these proteins and implicitly the target cells, which may now be recognized by spectral^4^ or temporal^3^^,^^5^^–^^7^^,^^18^^,^^19^ unmixing algorithms. The laser pulses used to facilitate temporal unmixing in OA imaging induce systematic and repeatable variations in the optical contrast provided by the rsOAPs that is distinguishable from the constant intrinsic contrast. This enables the detection of transfected cells against a background of natural chromophores. As the proteins are produced by cells expressing the reporter gene, it becomes feasible to monitor long-term changes in the cell population. Moreover, variations in the optical contrast provided by these proteins depend on the local fluence, a feature that has been utilized to account for optical attenuation and spectral coloring.^20^

The detection of rsOAPs has been demonstrated using differential OA imaging^3^^,^^5^ and temporal unmixing.^6^ In addition, a random forest algorithm and support vector machine (SVM) have been applied to features extracted via both differential imaging and temporal unmixing, which reported a binary semantic segmentation accuracy of 96% using a dataset of three mice, with histological reference.^7^ A limitation of this prior study is that pixels from the same mice were sampled for both training and validation purposes, meaning training and validation samples were not statistically independent, potentially leading to biased performance estimates. Moreover, histological reference data are limited by cost, experimental uncertainties (which can lead to label noise), and relatively small sample sizes compared with synthetic data. These methods can be used to visualize rsOAPs but fall short of quantifying the spatial concentration of the proteins or the target cells and do not utilize spatial information when making predictions. It has since been shown that when the intrinsic optical properties of a sample are known, it is possible to recover the concentration of different species of rsOAPs simultaneously^21^ by assuming the spatially dependent fluence does not vary between laser pulses. This model-based unmixing approach leverages limited spatial information through the use of total variation regularization, making it more robust to noise. However, in practice, the optical properties are usually unknown, and the light transmitted through rsOAPs varies with their optical absorption, causing the fluence to vary between laser pulses, while also obscuring rsOAPs at greater depths.^7^ In other words, spectral coloring is time-dependent when imaging rsOAPs with light. This effect, which occurs as shadowing,^5^ can lead to inaccuracies in unmixing if it is not accounted for, which requires solving the ill-posed and nonlinear optical inverse problem of quantitative OA imaging.^22^ Deep learning (DL) can be used to tackle such tasks and has the potential to significantly enhance the sensitivity of OA imaging for detecting rsOAPs, as well as its ability to quantify their spatial concentration when the intrinsic optical properties of intervening tissue are unknown. Unlike classical machine learning (ML) approaches, DL can leverage spatial information, such as potentially high correlation between nearby pixels, to improve their predictions, reduce noise sensitivity, and account for image reconstruction artifacts and spectral coloring.

In recent years, DL has achieved promising results in OA imaging applications, which include quantifying the concentration of certain chromophores,^23^^,^^24^ fluence correction,^25^ semantic segmentation,^26^ and image reconstruction.^27^^,^^28^ The usage of DL in OA image reconstruction is subdivided into direct image reconstruction,^28^ and DL-enhanced model-based reconstruction,^27^ with the latter usually leading to better results. The majority of studies utilized variants of the U-Net architecture^29^ and reported good results using synthetic data. Currently, the only synthetic OA imaging dataset containing rsOAPs does not take into account acoustic processes^21^ and assumes that the fluence is time-invariant so does not take into account shadowing.

In this study, we explored the application of DL in improving the sensitivity of the detection and quantification of rsOAPs with OA imaging. To address the need for large-scale training datasets, a 3D simulation framework was developed to generate labeled OA images of the proteins. This framework includes both optical and acoustic processes, as well as shadowing effects resulting from the time-varying optical fluence. As a result, we generated a synthetic dataset, consisting of 629 digital samples containing rsOAPs, that serves as a tool for developing methods of detecting rsOAPs in OA imaging. Although synthetic data may not be fully representative of experimental data, it does not suffer from label noise and is easier to produce in large quantities, which is required for DL training sets. We used this dataset to compare the performance of several well-established tree-ensemble and neural network models in two tasks related to CAR T-cell detection: binary semantic segmentation, and pixel-level prediction of the concentration of the rsOAPs. In contrast to prior model-based unmixing approaches^21^ for rsOAP quantification, which require knowledge of the optical properties, the present study focuses on data-driven inference from reconstructed OA images using supervised ML and DL models.

## 2 Methodology

### 2.1 Data Generation

**Fig. 1:**
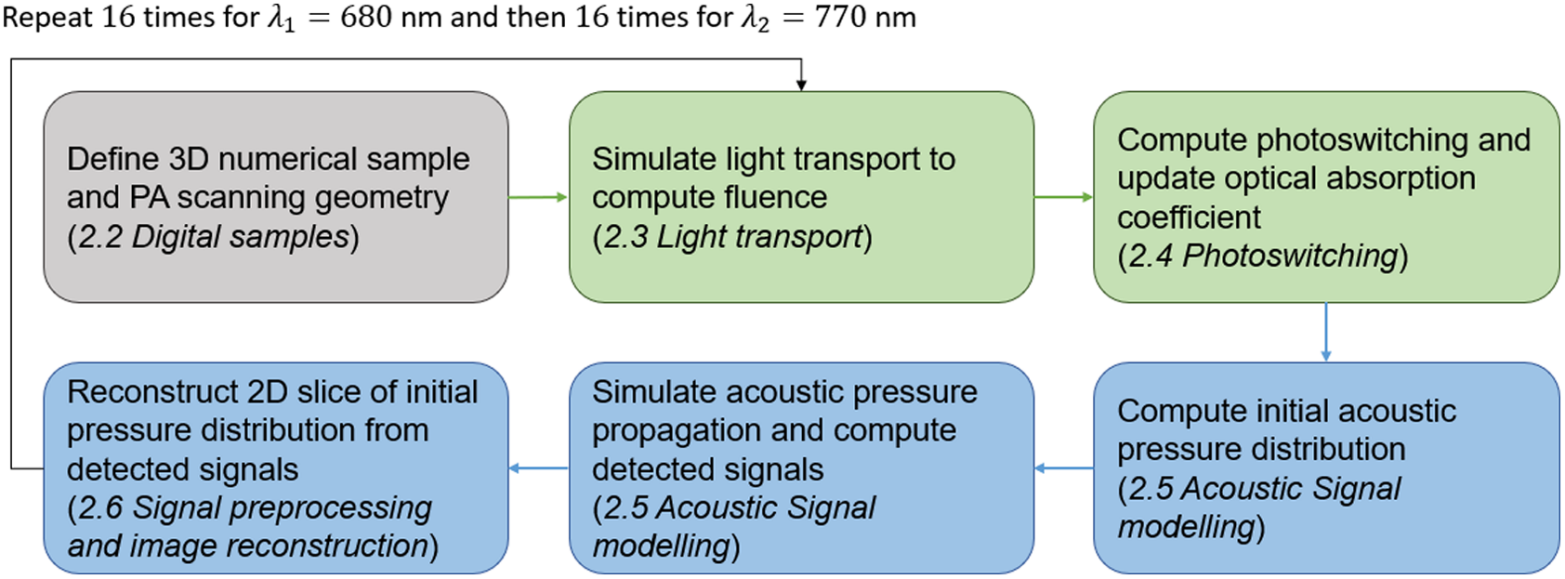
Workflow used to generate synthetic optoacoustic (OA) images of a sample containing reversibly switchable optoacoustic proteins (rsOAPs), which involves modeling of the optical processes (in green) followed by the acoustic processes (in blue). Here, $\lambda_{1}=680\;\mathrm{nm}$ and $\lambda_{2}=770\;\mathrm{nm}$ are the two wavelengths used to induce photoswitching in the proteins in this study. The relevant section in the paper is indicated in each box.

To generate the synthetic dataset, the physical processes behind laser pulse transport, the rsOAP state transition, OA signal generation and propagation, detection, and image formation were taken into account. Existing numerical techniques were used to model light and OA signal transport and to reconstruct OA images, whereas a model was developed to simulate the time-varying optical contrast provided by the rsOAPs. A flow chart of this data generation methodology, along with the relevant section number for each step, is presented in Fig. 1.

### 2.2 Digital Samples

As in preclinical multispectral optoacoustic tomography (MSOT) studies,^24^^,^^30^ inhomogeneous 3D cylindrical samples were considered. A total of 629 digital samples were generated overall. Each sample had a radius of 13 mm and was characterized by the intrinsic absorption $\mu_{a}^{\left(b\right)}$ and scattering $\mu_{s}^{\left(b\right)}$ coefficients of the background, a homogeneous anisotropy factor of g=0.9, and refractive index of $n_{\mathrm{RI}}=1.33$. Optical properties were considered at two wavelengths, $\lambda_{1}=680\;\mathrm{nm}$ and $\lambda_{2}=770\;\mathrm{nm}$, which are the wavelengths used in experimental OA imaging studies to both image the rsOAPs and induce their photoswitching.^7^ In this study, optical absorption due to rsOAPs or CAR T-cells was considered to be additive, rather than fractional, because the proteins are independent absorbers and because infiltrating CAR T-cells displace a negligible volume of intrinsic absorbers. Typical tissue cell density is orders of magnitude larger than infiltrating immune cells, and because rsOAPs or CAR T-cells are relatively small scatterers compared with other scatterers in tissue such as collagen fibers, the change in optical scattering induced by rsOAPs or CAR T-cells was neglected.

A random number, from 0 up to and including 8, nonoverlapping spherical inhomogeneities were fully embedded within each sample, with their centers in the central radial slice, which also contains the imaging plane. Each inhomogeneity had a randomly chosen radius $R_{t}$, which was sampled from a uniform distribution given in Table 1. These inhomogeneities were considered to represent: (i) regions of CAR T-cell accumulation in the homogeneous background, with the total absorption coefficient $\mu_{a}=\mu_{a}^{\left(b\right)}+\mu_{a}^{\left(p\right)}$, and scattering coefficient $\mu_{s}=\mu_{s}^{\left(b\right)}$, where $\mu_{a}^{\left(p\right)}$ is the extrinsic absorption coefficient due to the rsOAPs; (ii) tumors without CAR T-cells, with $\mu_{a}=\mu_{a}^{\left(b\right)}+\delta \mu_{a}^{\left(t\right)}$, and $\mu_{s}=\mu_{s}^{\left(t\right)}$, where $\delta \mu_{a}^{\left(t\right)}$ is the additional intrinsic absorption coefficient relative to the background due to tumors; and (iii) tumors containing CAR T-cells, with $\mu_{a}=\mu_{a}^{\left(b\right)}+\delta \mu_{a}^{\left(t\right)}+\mu_{a}^{\left(p\right)}$, and $\mu_{s}=\mu_{s}^{\left(t\right)}$.

**Table 1:** Parameters used to procedurally generate digital samples for this study. U(a,b) denotes a uniform distribution bounded by a and b, and N(a,b) a normal distribution with mean a and variance b. See Sec. 2.4 for explanation of absorption coefficient due to proteins $\mu_{a}^{\left(p\right)}$.

Parameter name	Symbol	Modeled as
Number of inhomogeneities	N	U({0,1,…,8})
Inhomogeneity radius	$R_{t}$	U(2,5) mm
Absorption coefficient (background)	$\mu_{a}^{\left(b\right)}$	$N(1.1\times 10^{-2}\;{\mathrm{cm}}^{-1},2.25\times 10^{-6}\;{\mathrm{cm}}^{-2})$
Absorption coefficient	$\delta \mu_{\text{a}}^{\left(t\right)}$	$\left(1-(r_{t}/R_{t}{)}^{2})N(1.1\times 10^{-2}\;{\mathrm{cm}}^{-1},2.25\times 10^{-6}\;{\mathrm{cm}}^{-2}\right)$,
(inhomogeneities)		where $r_{\text{t}}$ is the distance from the center of the inhomogeneity
Absorption coefficient (proteins)	$\mu_{\text{a}}^{\left(p\right)}$	$\varepsilon_{\mathrm{Pr}}c_{\mathrm{Pr}}+\varepsilon_{\mathrm{Pfr}}c_{\mathrm{Pfr}}$
Scattering coefficient (background)	$\mu_{\text{s}}^{\left(b\right)}$	$N(7\;{\mathrm{cm}}^{-1},0.49\;{\mathrm{cm}}^{-2})$
Scattering coefficient (inhomogeneities)	$\mu_{\text{s}}^{\text{(t)}}$	$N(15\;{\mathrm{cm}}^{-1},4\;{\mathrm{cm}}^{-2})$
Total protein concentration	$c_{\mathrm{tot}}$	$N(4\times 10^{-8}\;M,4\times 10^{-16}\;M^{2})$

The sample was considered a homogeneous, lossless acoustic medium, and the speed of sound within the sample was $1500\;{\mathrm{ms}}^{-1}$. The parameters $\mu_{a}^{\left(b\right)}$ and $\mu_{s}^{\left(b\right)}$ were sampled separately for each sample at each of the two imaging wavelengths from normal distributions given in Table 1. The parameters $\delta \mu_{a}^{\left(t\right)}$, and $\mu_{s}^{\left(t\right)}$ were sampled separately for each inhomogeneity at each of the two imaging wavelengths from normal distributions given in Table 1, and so, this model does not assume any correlation between these parameters across wavelengths. The distribution of rsOAPs was considered uniform across a given region containing CAR T-cells, and the concentration $c_{\mathrm{tot}}$ was also sampled from a normal distribution given in Table 1.

**Fig. 2:**
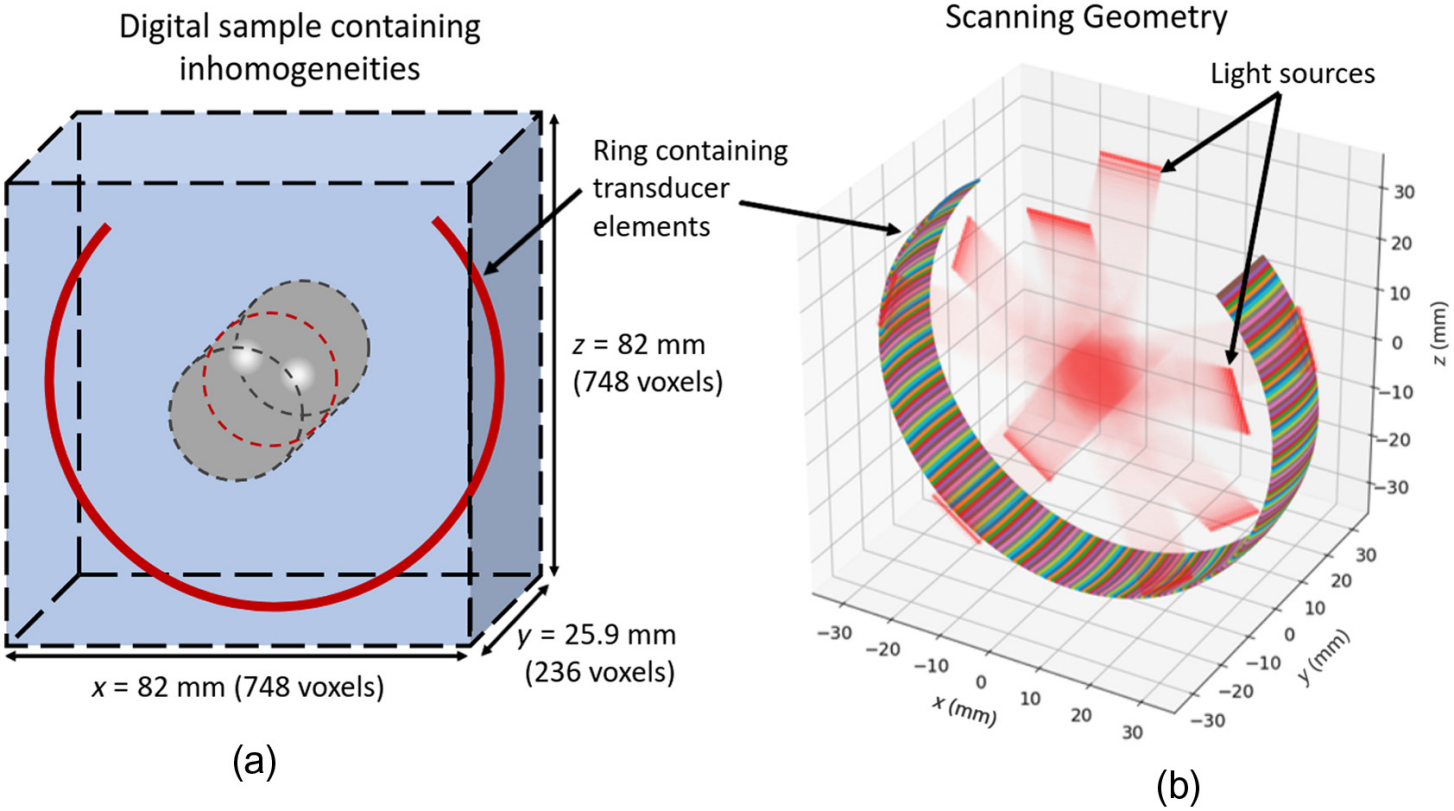
(a) Illustration of the simulation domain and a cylindrical sample containing spherical inhomogeneities centered in the central radial slice. (b) 3D scanning model based on the multispectral optoacoustic tomography (MSOT) in-Vision 256™ system (iThera GmbH, Munich).^30^ The light source geometry is shown in red, and individual elements (curved in the y-direction for out-of-plane focusing) in the ultrasound transducer array are positioned on the ring.

The sample, light source, and detector are illustrated in Fig. 2 and were discretized into 748 by 236 by 748 cubic voxels of side length 0.110 mm. This allowed the simulation to support acoustic waves of frequencies up to 6.84 MHz without exceeding 24 GB of VRAM on a GPU.

### 2.3 Light Transport

The 3D model of the OA scanning geometry was based on the MSOT in-Vision 256™ system (iThera GmbH, Munich).^30^ The illumination of each sample was simulated in 3D using the open-source GPU-accelerated Monte Carlo eXtreme (MCX) software written in CUDA^31^ by simulating $10^{8}$ photons per illumination with the implementation of the MSOT light source geometry from SIMPA.^24^^,^^32^ Specifically, five pairs of rectangular light sources of size $12.4\times 0.2\;{\mathrm{mm}}^{2}$ are positioned around the sample, covering 270 deg in azimuth, and focused toward its central radial slice. Figure 2 illustrates the sample and scanning geometry. Each sample was illuminated by 16 pulses sequentially at $\lambda_{1}=680\;\mathrm{nm}$, followed by 16 pulses sequentially at $\lambda_{2}=770\;\mathrm{nm}$, generating an image for each illumination, resulting in a temporal sequence of 32 images per sample, as shown in the flow chart in Fig. 1.

MCX simulates the normalized fluence at every point r in the computational domain for each laser pulse i. This is then multiplied by the total energy $E_{i}$ delivered by the light source during the pulse, resulting in the fluence $\Phi_{i}$. The MSOT measures $E_{i}$ while performing experimental scans and uses it for a laser energy correction, where the measured OA signals are divided by $E_{i}$ before the images are reconstructed. In this study, the energy of the laser pulses was modeled as $E_{i}(\lambda_{1})=N(58.01\;\mathrm{mJ},3.31\;{\mathrm{mJ}}^{2})$ and $E_{i}(\lambda_{2})=N(70.07\;\mathrm{mJ},0.56\;{\mathrm{mJ}}^{2})$, with the choice of the mean energy and variance guided by experimental data from the Institute of Cancer Research (London, United Kingdom). An energy correction was performed on the simulated OA signals before the images were reconstructed.

### 2.4 Photoswitching

**Fig. 3:**
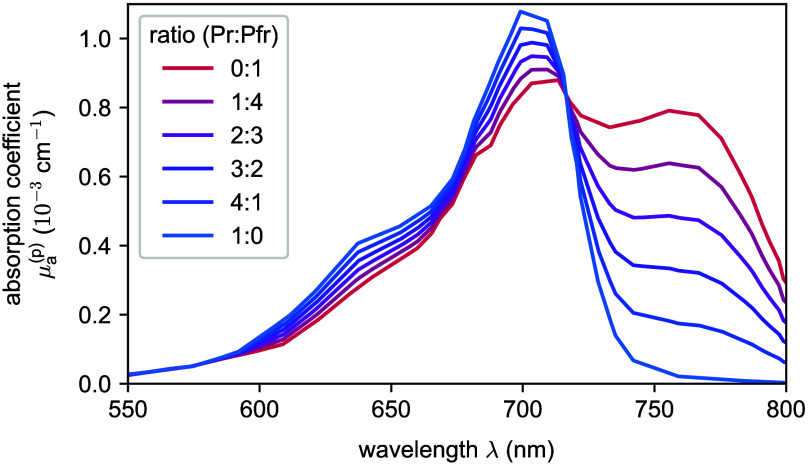
Simulated absorption coefficient $\mu_{a}^{\left(p\right)}$ of the reversibly switchable optoacoustic protein (rsOAP) for various ratios of the red-absorbing (Pr) and far-red-absorbing (Pfr) concentrations, assuming a total protein concentration of $c_{\mathrm{tot}}=10^{-8}$ M.

The rsOAP considered in this study mimics the bacterial phytochrome modified from *Rhizobium etli*, ReBphP-PCM,^7^ which exists in two metastable absorbing states, the red-absorbing (Pr) and far-red-absorbing (Pfr) states.^7^^,^^18^ Under optical illumination, it undergoes a reversible photochemical transformation between the Pr and the Pfr states: optical pulses at wavelengths of 740 to 770 nm switch Pfr to Pr, and optical pulses at wavelengths of 660 to 700 nm switch Pr to Pfr.^7^^,^^33^ Correspondingly, the absorption coefficient of the rsOAPs when under illumination with laser pulse i at time $t_{i}$ and wavelength λ is given by

$$\mu_{a}^{\left(p\right)}(r,\lambda ,t_{i})=\epsilon_{\mathrm{Pr}}(\lambda )c_{\mathrm{Pr}}(r,t_{i})+\epsilon_{\mathrm{Pfr}}(\lambda )c_{\mathrm{Pfr}}(r,t_{i}),$$ (1)

where $t_{i}=(i-1)\Delta t$, with i≥1 and Δt the time between pulses. $\epsilon_{\mathrm{Pr}}$ and $\epsilon_{\mathrm{Pfr}}$ are the molar absorption coefficients, and $c_{\mathrm{Pr}}$ and $c_{\mathrm{Pfr}}$ are the molar concentrations corresponding to the Pr and Pfr states, respectively. Figure 3 presents $\mu_{a}^{\left(p\right)}$ for various ratios of $c_{\mathrm{Pr}}$ and $c_{\mathrm{Pfr}}$, which was computed using data for $\epsilon_{\mathrm{Pr}}$ and $\epsilon_{\mathrm{Pfr}}$ from^7^ and Eq. (1).

The fluence $\Phi_{i}$ computed for the pulse at $t_{i}$ was used to update $c_{\mathrm{Pr}}$ and $c_{\mathrm{Pfr}}$ before illumination with the next pulse at $t_{i+1}$ as

$$c_{\mathrm{Pr}}(r,\lambda ,t_{i+1})=\alpha (r,\lambda )+[c_{\mathrm{Pr}}(r,\lambda ,t_{i})-\alpha (r,\lambda )]\exp[-\beta (\lambda )\Phi_{i}(r,\lambda )].$$ (2)

Equation (2) was obtained by modeling the rsOAPs as a two-state system, with instantaneous transitions between states driven by laser pulses at the two wavelengths $\lambda_{1}$ and $\lambda_{2}$. The derivation of Eq. (2), as well as the definition of the parameters α and β, is given in Sec. 6.1 in the Appendix. We note that Eq. (2) predicts that the optical contrast provided by the photoswitching proteins has a pulse number (time) independent component and a component that decays exponentially with the number of laser pulses applied at a given wavelength, which is in agreement with observations from experimental studies.^6^^,^^34^ The concentration of rsOAPs in the Pfr state is obtained as $c_{\mathrm{Pfr}}=c_{\mathrm{tot}}-c_{\mathrm{Pr}}$.

### 2.5 Acoustic Signal Modeling

The initial acoustic pressure distribution generated at a point r in the sample by a laser pulse at $t_{i}$ was calculated as

$$p_{0}(r,\lambda ,t_{i})=\Phi_{i}(r,\lambda ,t_{i})\mu_{a}(r,\lambda ,t_{i}),$$ (3)

where the Grüneisen parameter and the thermalization efficiency^35^ were considered equal to 1 as in other numerical OA imaging studies.^23^^,^^24^ The pressure as a function of time across the surface of the detectors for each image acquisition was computed by solving the time-dependent wave equation obeyed by the pressure with $p_{0}$ as the initial value boundary condition. The equation was solved in 3D using the compiled CUDA code and Python implementation^36^ of the k-Wave toolbox.^37^ The 3D model of the MSOT ultrasonic transducer array was adopted from Ref. 38 and reimplemented from MATLAB (MathWorks, Natick, Massachusetts, United States) into Python for this study, ensuring that all software requirements are open source. The MSOT detector array consists of 256 elements, which are spherically curved in the y-direction to provide toroidal focusing with a fixed center of curvature at the center of the ring and provide 270 deg angular coverage, as shown in Fig. 2.

### 2.6 Signal Preprocessing and Image Reconstruction

The primary factor in the detectability of rsOAPs is the SNR of the time-varying contrast between successive reconstructed images as this will determine whether an unmixing or ML algorithm is able to distinguish photoswitching from noise. The OA signal amplitude is directly proportional to the Grüneisen parameter, which is <1 for all tissues.^39^ However, when the Grüneisen parameter is homogeneous and there is a fixed amount of additive noise, assigning and simulating a lower value only have the effect of reducing the SNR. In this study, the SNR is varied by adjusting the amount of noise added to the detected OA signals prior to reconstruction, assuming that the Grüneisen parameter is 1. Three datasets were constructed from the simulated OA signals, each with a different level of additive Gaussian noise: no noise (dataset 1), noise distributed as $N\{0\;\mathrm{Pa},4\;{\mathrm{Pa}}^{2}\}$ (dataset 2, with ${\mathrm{SNR}}_{\mathrm{dB}}=18.4\;\mathrm{dB}$), and noise distributed as $N\{0\;\mathrm{Pa},36\;{\mathrm{Pa}}^{2}\}$ (dataset 3, with ${\mathrm{SNR}}_{\mathrm{dB}}=8.8\;\mathrm{dB}$). The ${\mathrm{SNR}}_{\mathrm{dB}}$ of dataset 2 is roughly equivalent to the ${\mathrm{SNR}}_{\mathrm{dB}}$ in experimental measurements,^40^ whereas dataset 3 represents a high-noise scenario. These ${\mathrm{SNR}}_{\mathrm{dB}}$ values for datasets 2 and 3 were calculated as

$${\mathrm{SNR}}_{\mathrm{dB}}=20\;\log_{10}(\frac{{\mathrm{RMS}}_{\text{signal}}}{{\mathrm{RMS}}_{\text{noise}}}),$$ (4)

where ${\mathrm{RMS}}_{\text{signal}}$ and ${\mathrm{RMS}}_{\text{noise}}$ are the root mean square values of the signal and noise, respectively. ${\mathrm{RMS}}_{\text{signal}}$ was computed using the simulated pressure as a function of time across all detectors, for all samples, image acquisitions, and wavelengths. Small signals originating from outside the samples were excluded from the RMS calculation to prevent them from artificially lowering the ${\mathrm{RMS}}_{\text{signal}}$ estimate, resulting in ${\mathrm{RMS}}_{\text{signal}}=16.6\;\mathrm{Pa}$. No ${\mathrm{SNR}}_{\mathrm{dB}}$ value is reported for dataset 1 because it does not contain any noise.

**Fig. 4:**
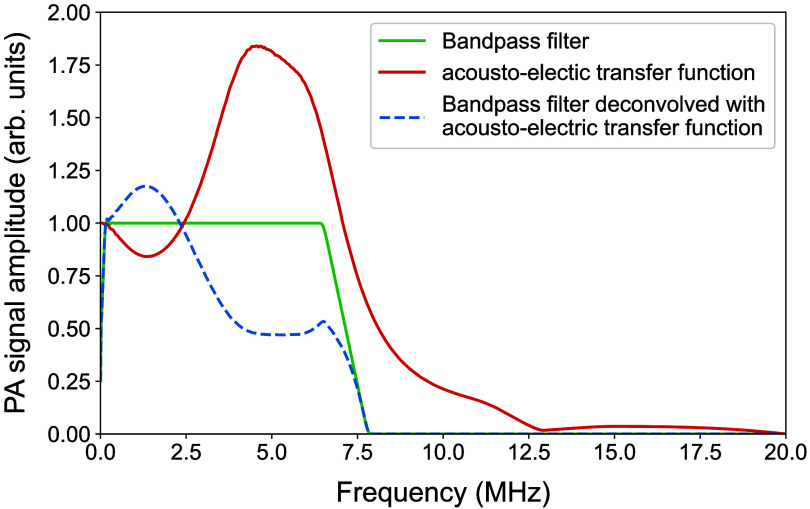
Multispectral optoacoustic tomography (MSOT) detector acousto-electric transfer function, bandpass filter, and bandpass filter deconvolved with acousto-electric transfer function.

A detector acousto-electric transfer function was used to simulate the band-limited response of the MSOT transducer array in the synthetic datasets by convolving it with the detected OA signals. After this step, a bandpass filter (50 kHz to 6.5 MHz) implemented with the PATATO toolkit^41^ was applied to the two datasets with noise (datasets 2 and 3). As a method of deconvolution with the applied acousto-electric transfer function, the bandpass filter was divided by the previously applied acousto-electric transfer function in the frequency domain, before being applied to the noisy detected OA signals. The bandpass filter and acousto-electric transfer function (obtained from experimental data from the Institute of Cancer Research) used in this study are presented in Fig. 4.

The initial acoustic pressure distribution $p_{0}$ was reconstructed in 2D for the central slices of the samples using iterative adjoint model-based reconstructions^42^ implemented with the k-Wave-Python library and the compiled k-Wave CUDA code.^36^^,^^37^ Five iterations were used with a positivity constraint that maps all negative values in the reconstructed images to zero; this image reconstruction method, based on the data in this study, resulted in reconstructions with a substantially lower error than a universal back-projection algorithm,^43^ as measured by the root mean squared error (RMSE) between the reconstructed images and the ground truth. A qualitative assessment indicated that RMSE improvements plateaued after five iterations. The band-limited acoustic response and finite detector aperture introduce a system point spread function, which results in boundary blurring and effective partial-volume–like behavior in the reconstructed images.

### 2.7 Machine Learning Models

Several well-established ML models^44^^,^^45^ spanning distinct methodological categories were considered for two tasks relevant to rsOAP detection: binary semantic segmentation, and pixel-level prediction (regression) of the rsOAP concentrations from the temporal sequences of synthetic images. Although concentration prediction provides richer output, binary semantic segmentation was also evaluated because binary masks are typically easier to obtain than accurate concentration labels in experimental settings, and binary predictions may be less sensitive to scaling shifts (e.g., changes in the effective Grüneisen parameter) between training and inference.

For the ML experiments, two tree-ensemble models, random forest (RF)^46^ and extreme gradient boosted trees (XGB),^47^ were selected because they provide strong baselines for learning from engineered temporal features, and RF has been used in a prior study on rsOAPs.^7^ These models were both implemented using the Xgboost Python library.^47^ Support vector machine (SVM) is another widely used baseline, which was used in the same previous study as RF.^7^ However, the memory complexity of SVM scales quadratically with the number of training examples at best, and therefore, it was not feasible to apply SVM to the training data sets of 3 million pixels in this study. Four neural network models implemented using the PyTorch framework^48^ were also used. A multilayer perceptron (MLP) was chosen to serve as a neural baseline without explicit spatial modeling or a large number of parameters. The MLP consists of three hidden linear layers of size 512 separated by batch normalization layers,^49^ ReLU activation functions, and dropout layers.^50^ Convolutional neural networks (CNNs), U-Net-ResNet101^29^ and DeepLabV3-ResNet101,^51^ were selected for their ability to utilize local spatial context and because of the success of U-Net in previous OA imaging studies.^25^^,^^38^ Both CNNs were constructed using a ResNet-101^52^ backbone pretrained for image classification on the ImageNet dataset.^53^ Last, the transformer-based architecture SegFormer-B5^54^ pretrained for semantic segmentation on the ADE20K dataset^55^ was included because of its ability to model global spatial dependencies.

### 2.8 Image Processing and Feature Extraction

It was found that the tree-ensemble methods and MLP were unable to detect the rsOAPs directly from the reconstructed OA signal as a function of the pulse number. So, to apply these models, 12 temporal features were extracted from each pixel using temporal unmixing.^6^ This consists of fitting the objective function,

$$(A,k,b)=\underset{\left(A,k,b\right)}{\mathrm{argmin}}\{\underset{i}{\sum }{\left(p_{0}^{\left(i\right)}-(b+Ae^{-ki})\right)}^{2}\},$$ (5)

for each excitation wavelength. Here $p_{0}^{\left(i\right)}$ is the reconstructed OA signal for pulse i, A is the initial value of the time-varying reconstructed signal, k is the decay constant, and b is the time-independent reconstructed signal. This objective function was defined based on the form of Eq. (2), and was fit using a Levenberg–Marquardt algorithm implemented by the SciPy library.^56^

The parameters A, k, b and the coefficient of determination $R_{f}^{2}$ provided 4 features per wavelength. Additionally, 2 more features per wavelength were obtained by taking the range image $\mathrm{RI}(p_{0}^{\left(i\right)})=\max(p_{0}^{\left(i\right)})-\min(p_{0}^{\left(i\right)})$, and the difference image $\mathrm{DI}(p_{0}^{\left(i\right)})=p_{0}^{\left(1\right)}-p_{0}^{\left(16\right)}$.^6^ When rsOAPs are present, the resulting time-varying optical contrast is expected to produce a good decay curve fit ($R_{f}^{2}>0.7$), and A, RI and DI are expected to be positive and to take similar values (positively correlated). If the local fluence is constant between successive images, using Eq. (2), it can be shown that the decay constant is directly proportional to the fluence. However, the decay constant k obtained from temporal unmixing is sensitive to noise and variations in the fluence between successive images caused by shadowing, so it cannot reliably be used to determine the local fluence in this case. Where shadowing occurs, A and DI are expected to be negative, and RI is expected to be positive (negatively correlated). Last, when the SNR and/or protein concentration is too low, the time-varying component of $p_{0}^{\left(i\right)}$ is dominated by noise, so the fit does not converge (low $R_{f}^{2}$), A, k, and b usually diverge to meaningless values, whereas RI and DI are random and uncorrelated.

All features for pixels outside the sample were set to zero. The 12 extracted features were thresholded to ensure that their variance was not dominated by instances where the fit from Eq. (5) failed to converge. A and b were thresholded between $\max(p_{0}^{\left(i\right)})$ and $-\max(p_{0}^{\left(i\right)})$, and k between −0.5 and 0.5. Negative values of k indicate exponential growth rather than decay and were obtained only when the fit failed to converge, which is an indication that the protein concentration in the pixel is close to zero. The feature images were initially noisy, due to pixels where the fit from Eq. (5) failed to converge, and the Gaussian noise that was added to the signals prior to image reconstruction. To counteract this, a 3×3 kernel median filter was applied to smooth images of the features A, k, and b. The $R_{f}^{2}$ values for the fit were calculated after thresholding and filtering A, k, and b.

### 2.9 Machine Learning Experiment Setup

U-Net-ResNet101, DeepLabV3-ResNet101, and SegFormer-B5 were trained and evaluated using both the extracted feature images and the raw temporal sequences of images separately. This is because CNN and transformer models can learn to extract features directly from image data, so it is not always necessary to extract engineered features to achieve good performance. The results are only presented for RF, XGB, and MLP when trained using the pixel-level features from the feature images because these models failed to classify the proteins from the raw pixel-level temporal sequences. The effect of noise in the input data on the performance of the models was studied by training and evaluating each model separately on the three datasets with different noise levels described in Sec. 2.6. Before being used with ML models, the temporal image sequences in each dataset were normalized by

$$p_{0,\text{normalised}}^{\left(i\right)}=\frac{p_{0}^{\left(i\right)}-\min(p_{0}^{\left(i),(\text{all}\right)})}{\max(p_{0}^{\left(i),(\text{all}\right)})-\min(p_{0}^{\left(i),(\text{all}\right)})},$$ (6)

where $\max(p_{0}^{\left(i),(\text{all}\right)})$ and $\min(p_{0}^{\left(i),(\text{all}\right)})$ are the maximum and minimum values, respectively, of $p_{0}^{\left(i\right)}$ across all samples, wavelengths, pixels, and time steps $t_{i}$. Similarly, each feature $f_{\left(j\right)}$ was normalized independently of other features with respect to the entire dataset by

$$f_{j,\text{normalised}}=\frac{f_{j}-\min(f_{j}^{\left(\text{all}\right)})}{\max(f_{j}^{\left(\text{all}\right)})-\min(f_{j}^{\left(\text{all}\right)})},$$ (7)

where $\max(f_{j}^{\left(\text{all}\right)})$ and $\min(f_{j}^{\left(\text{all}\right)})$ are the maximum and minimum values, respectively, of feature $f_{j}$ across all samples and pixels. These normalization statistics were only computed using the training splits to avoid data leakage.

As the encoders of U-Net-ResNet101, DeepLabV3-ResNet101, and SegFormer-B5 were pretrained using three channel (RGB) images, the first layer of each network was modified to accept either 32- or 12-channel inputs, depending on whether the model was trained using the temporal images or the extracted feature images, respectively. This was achieved by summing the weights and biases of RGB kernels along the channel axis and then duplicating them to the desired number of channels.

For DeepLabV3-ResNet101 and SegFormer-B5, the output predictions were, by default, 1/8 and 1/4 of the scale of the input images, respectively. To avoid performance being inflated by the removal of high frequency information, and partial-volume effects, the predictions were evaluated at full image resolution. For binary semantic segmentation, nearest-neighbor interpolation was sufficient to obtain prediction images that were the same size as the input images. For pixel-level prediction, it was necessary to upsample the outputs by adding a bilinear interpolation upsampling layer with a factor of 2, followed by a convolutional layer with a 3 by 3 kernel in succession until the output dimensions matched the input. Thus, two upsampling layers and convolutional layers were added to SegFormer-B5, and three were added to DeepLabV3-ResNet101. For both models, this resulted in smoother predictions and lower losses on the validation dataset.

### 2.10 Model Training and Evaluation

Of the 629 samples, 504 were used for training, 63 for validation, and 62 for testing. The three noise levels were treated as separate datasets. The same 63 and 62 samples were used for validation and testing, respectively, when constructing the training, validation, and testing splits for each dataset. All models were trained using an NVIDIA GeForce RTX 3090 GPU. Binary cross-entropy was used as the objective function for binary semantic segmentation, whereas the mean squared error was used for pixel-level prediction. Each deep neural network model was trained for 100 epochs using the Adam optimizer^57^ and a batch size of 16. A total of 100 epochs were chosen because a qualitative inspection of the loss curves concluded that the validation losses of the models stopped improving before this point. The learning rate for U-Net-ResNet101, DeepLabV3-ResNet101, and SegFormer-B5, was set to 0.001. The hyperparameters of RF, XGB, and MLP were optimized using the Optuna framework,^58^ with 100 trials per model, per task. In addition, each ML model was trained five times for each task and each variation of the input data. A different seed was used each time for any dropout layers or randomly initiated parameters, and the PyTorch flag to use deterministic CUDA algorithms was set to false, so the results were less affected by a specific seed or algorithm choices.

The performance of models trained for binary semantic segmentation was evaluated in terms of the intersection over union,

$$\mathrm{IoU}=\frac{\mathrm{TP}}{\mathrm{TP}+\mathrm{FP}+\mathrm{FN}},$$ (8)

sensitivity,

$$\text{sensitivity}=\frac{\mathrm{TP}}{\mathrm{TP}+\mathrm{FN}},$$ (9)

and specificity,

$$\text{specificity}=\frac{\mathrm{TN}}{\mathrm{TN}+\mathrm{FP}}.$$ (10)

IoU measures the overlap between predicted and ground-truth positive regions; sensitivity, also known as recall, measures the proportion of true positives correctly identified (true positive rate); and specificity measures the proportion of true negatives correctly identified (true negative rate). TP is the number of true positives, TN is the number of true negatives, FP is the number of false positives, and FN is the number of false negatives from the binary confusion matrix.^59^ In this study, IoU was preferred over Dice score because, unlike Dice score, IoU satisfies the triangle inequality, meaning it can be considered a proper metric. The models trained for regression were evaluated in terms of the root mean squared error (RMSE),

RMSE=1N∑k=1N(y^k−yk)2, (11)

and mean absolute error (MAE),

MAE=1N∑k=1N|y^k−yk|, (12)

where $y_{k}$ and y^k are the ground-truth and predicted concentrations at pixel k, and N is the number of pixels. These metrics were selected to reflect the clinical objectives of detecting CAR T-cell regions and quantifying their concentration. Sensitivity and specificity capture the trade-off between missed detections and false positives, whereas IoU measures spatial agreement. IoU, sensitivity, and specificity range from 0 to 1, with higher values representing better performance. MAE and RMSE provide measures of typical quantification error and are both given in units of M, but RMSE emphasizes large deviations more. To ensure that model performance in background regions does not obscure performance in inhomogeneities, each metric was computed per sample, for the inhomogeneities and background separately. Here, background refers to the region inside the cylindrical samples but outside of the inhomogeneities.

## 3 Results

### 3.1 Forward Model Validation

**Fig. 5:**
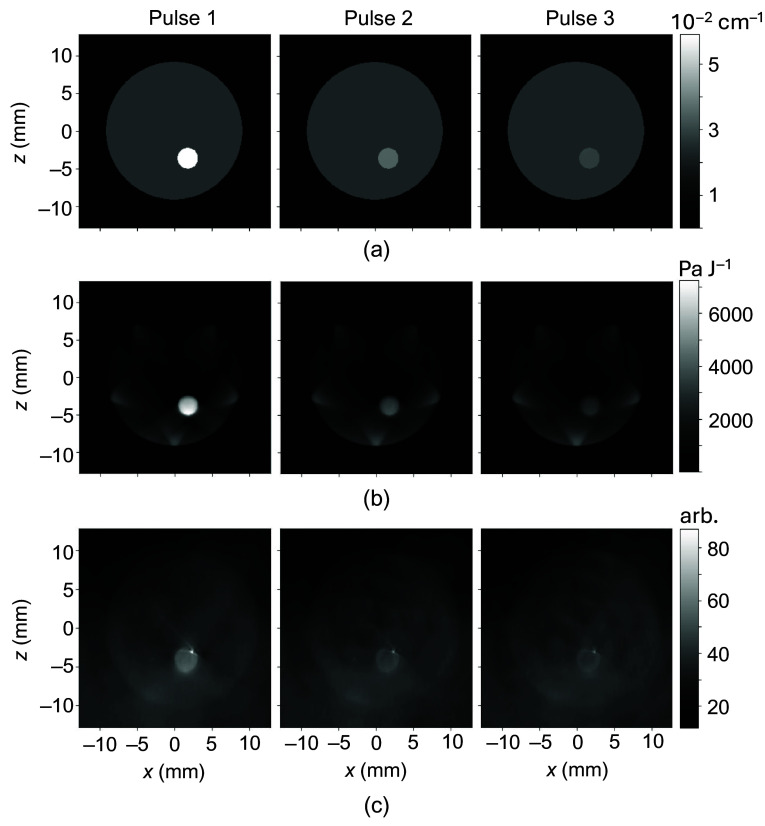
For three of the imaging pulses at 770 nm and the central slice of each phantom, (a) total absorption coefficient of a digital sample, (b) reconstructed initial pressure distribution of the digital sample, and (c) reconstructed initial pressure distribution of the experimental phantom.

**Fig. 6:**
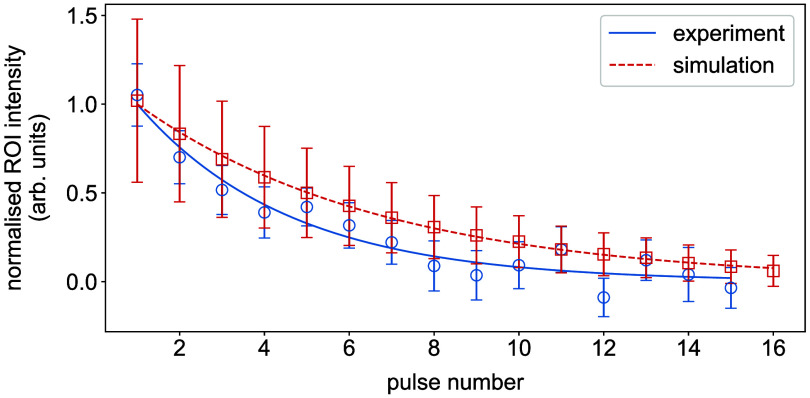
Normalized decay curves of the mean OA signal throughout the ROI in the images reconstructed from experimental (blue) and simulated (red) data. The experimental images were averaged over five cycles. The error bars show the standard deviations across the ROIs. The experimental phantom was imaged using 15 pulses per wavelength, whereas 16 pulses per wavelength were simulated.

To validate the photoswitching model described in Sec. 2.4, a comparison was made between simulated images of a sample generated as described in Sec. 2.2 and experimental OA images of a cylindrical phantom containing bacteria expressing the reporter gene. Figure 5(b) shows images of the simulated phantom at 770 nm reconstructed with the model-based reconstructions detailed in Sec. 2.6.^42^ Images of the experimental phantom at 770 nm, reconstructed using the back-projection algorithm in viewMSOT 4.0 and averaged over five cycles, are shown in Fig. 5(c). The region of interest (ROI) containing the proteins was segmented from the simulated and experimental images. Figure 6 presents decay curves fitted to the mean reconstructed OA signal across each ROI as a function of the pulse number using Eq. (5), with the parameters and corresponding coefficient of determination obtained from each fit given in Table 2.

**Table 2:** Parameters obtained by fitting Eq. (5) to the mean OA signal of the ROI from experimental and simulated images of the protein.

Parameter	Experiment	Simulation
Amplitude A	11.05 arb. unit	$3865\;\mathrm{Pa}\;J^{-1}$
Decay constant k	$0.2783\;{\text{pulses}}^{-1}$	$0.1705\;{\text{pulses}}^{-1}$
Time-independent contrast b	40.05 arb. unit	$1813\;\mathrm{Pa}\;J^{-1}$
Coefficient of determination $R^{2}$	0.9421	0.9988

### 3.2 Synthetic Data

**Fig. 7:**
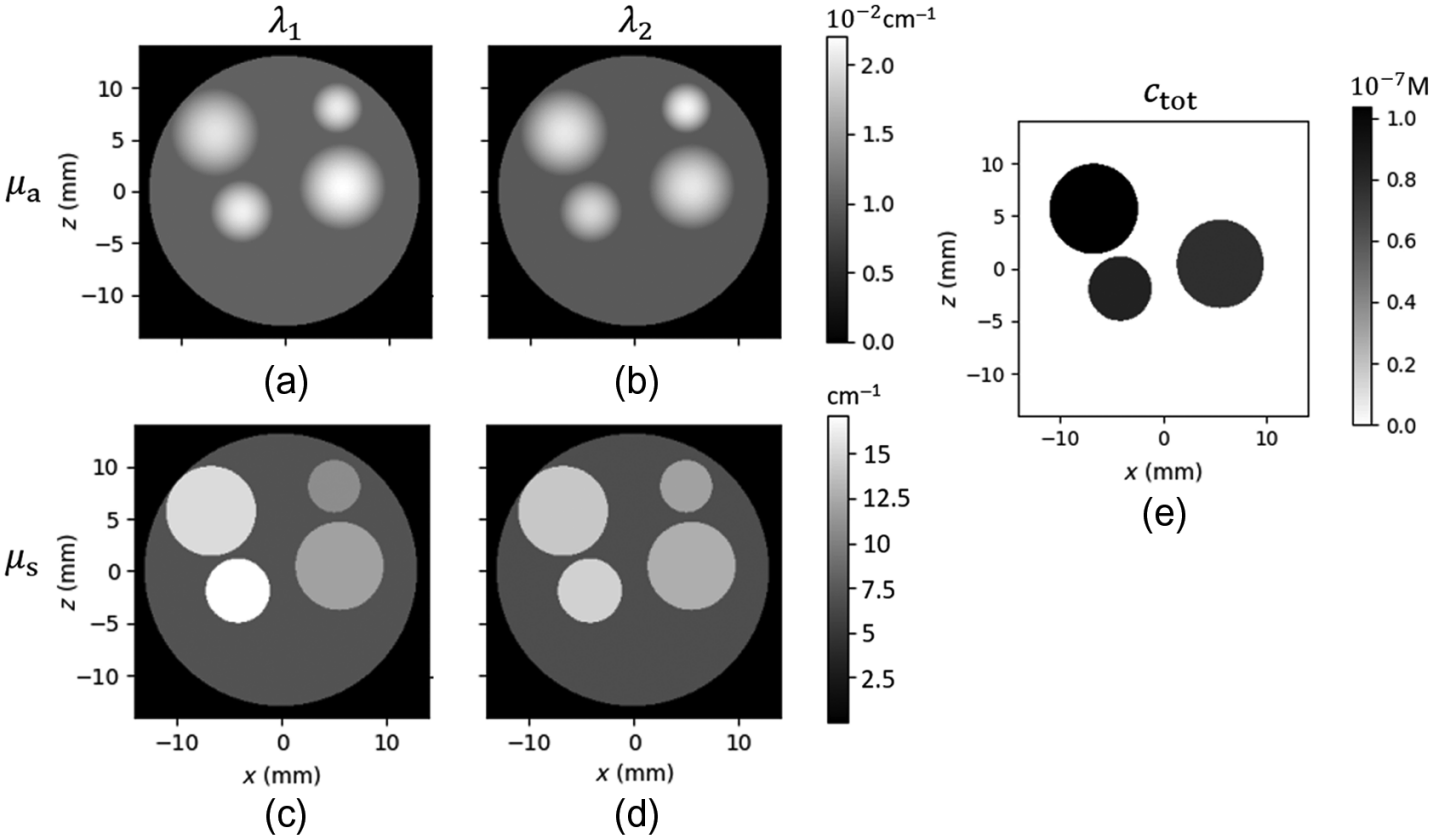
Intrinsic absorption coefficient $\mu_{a}$ (a)–(b), and scattering coefficient $\mu_{s}$ (c)–(d) at wavelengths $\lambda_{1}=680\;\mathrm{nm}$ and $\lambda_{2}=770\;\mathrm{nm}$, respectively, and reversibly switchable optoacoustic protein (rsOAP) concentration $c_{\mathrm{tot}}$ (e), in the central slice of a digital sample. In this example, three out of the four inhomogeneities contain rsOAPs.

**Fig. 8:**
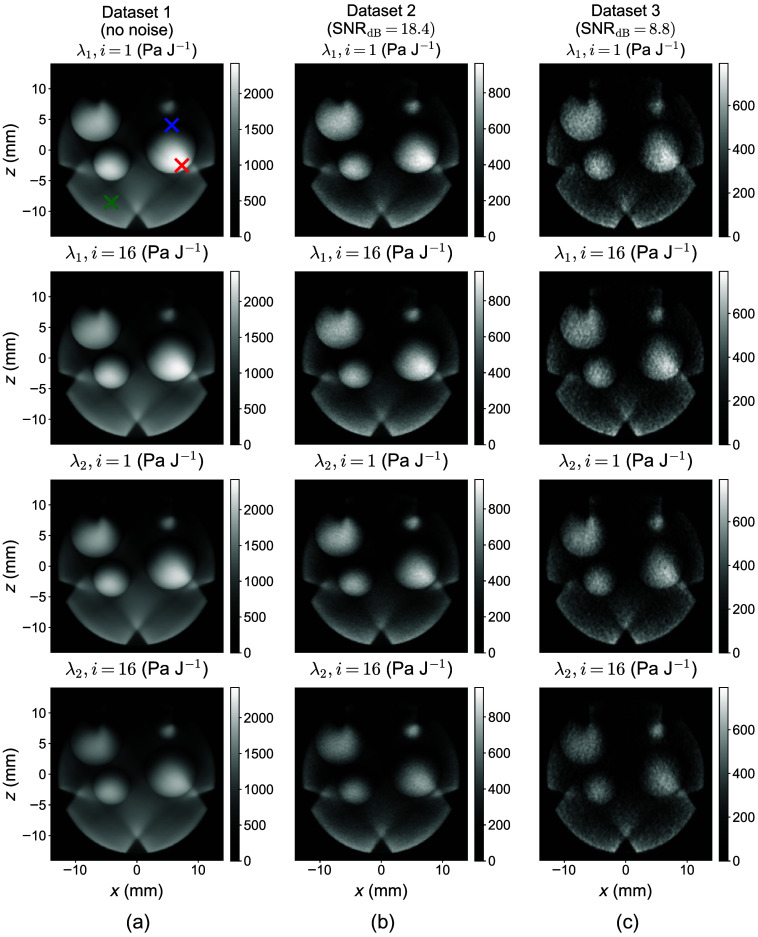
Laser-energy-corrected reconstructed images of the initial pressure $p_{0}$ for the digital sample shown in Fig. 7, for (a) dataset 1 with no noise in the data, (b) dataset 2 with signal-to-noise ratio ${\mathrm{SNR}}_{\mathrm{dB}}=18.4\;\mathrm{dB}$, and (c) dataset 3 with ${\mathrm{SNR}}_{\mathrm{dB}}=8.8\;\mathrm{dB}$, for (first row) first pulse at $\lambda_{1}$, (second row) last (16th) pulse at $\lambda_{1}$, (third row) first pulse at $\lambda_{2}$, (fourth row) last (16th) pulse at $\lambda_{2}$. Three pixels used to illustrate the reconstructed pixel value as a function of the pulse number are indicated in the top left image.

The central radial slice of one of the digital samples is shown in Fig. 7. Here, each spherical inhomogeneity has different intrinsic absorption and scattering. Three out of the four spherical inhomogeneities contain rsOAPs, representing regions where CAR T-cells have accumulated. Reconstructed images of $p_{0}$ for the digital sample from Fig. 7 are shown in Fig. 8 for the three datasets with different noise levels. The color bar units for the reconstructed images are $\mathrm{Pa}\;J^{-1}$, due to the laser energy correction. We note that these images present artifacts in the form of a weaker signal in the upper half of the sample resulting from the limited angular coverage of the detector. The effects of the nonuniform illumination of the sample are also visible in the form of bright triangles where the illumination paths overlap, and dark triangles outside the illumination paths close to the sample surface. In addition, image quality degrades as the noise in the measurement data increases, but the inhomogeneities can still be distinguished with high levels of noise. The greatest change in optical absorption coefficient (contrast) occurs between the first and last images at $\lambda_{2}$ when no noise was added, so it is easier to see photoswitching between these two images, but it is nevertheless very difficult to determine which of the four inhomogeneities contain rsOAPs without extracting features.

### 3.3 Feature Extraction

**Fig. 9:**
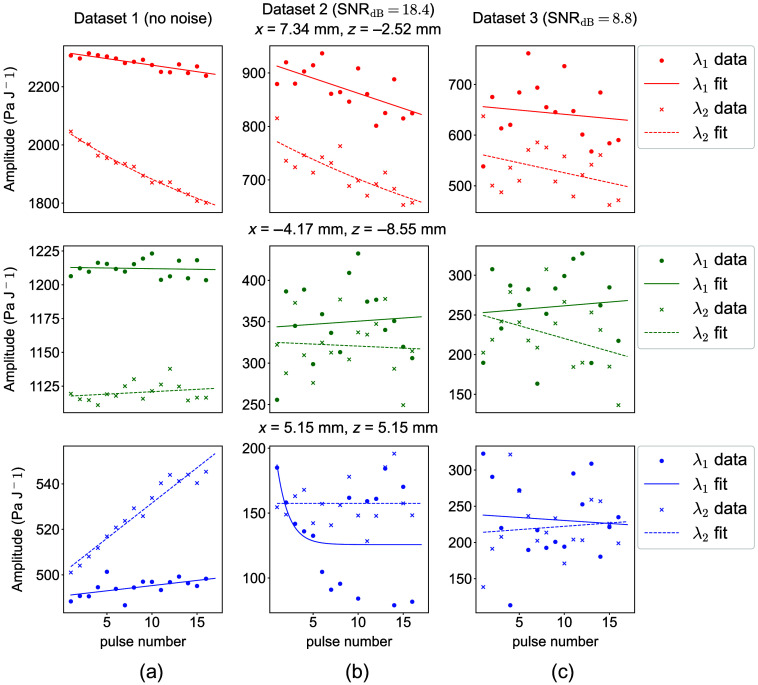
Laser-energy-corrected reconstructed initial pressure $p_{0}$ as a function of the pulse number fit to Eq. (5), for the pixels indicated in Fig. 8 located at (7.34,−2.52  mm) (top row), (−4.17,−8.55  mm) (middle row), and (5.70, 4.06 mm) (bottom row), for dataset 1 with no noise in the data (a), dataset 2 with signal-to-noise ratio ${\mathrm{SNR}}_{\mathrm{dB}}=18.4\;\mathrm{dB}$ (b), and dataset 3 with ${\mathrm{SNR}}_{\mathrm{dB}}=8.8\;\mathrm{dB}$ (c). The circular markers and solid lines represent the data and fitted decay curves for the first 16 pulses (at $\lambda_{1}=680\;\mathrm{nm}$), whereas cross markers and dashed lines represent the data and fitted decay curves for the last 16 pulses (at $\lambda_{2}=770\;\mathrm{nm}$). Imaging was performed with 16 pulses at $\lambda_{1}$, before imaging with 16 pulses at $\lambda_{2}$.

The reconstructed value of the initial pressure $p_{0}$ as a function of the pulse number fit to Eq. (5), for the 3 pixels indicated in Fig. 8, are shown in Fig. 9 for each dataset and wavelength. The pixel at (7.34,−2.52  mm) (indicated in red in Fig. 8) is located in a region of rsOAP accumulation. For no noise, and medium noise in the data (datasets 1 and 2), $p_{0}$ at this location decays with the number of applied pulses due to photoswitching. The change in $p_{0}$ (time-varying contrast) after each applied pulse is smaller when imaging at 680 nm compared with 770 nm because the change in $\mu_{a}^{\left(p\right)}$ at 680 nm is smaller when the rsOAPs switch, as seen in Fig. 3. The pixel at (−4.17,−8.55  mm) (indicated in green in Fig. 8) is located in the background of the sample and is well separated from regions containing rsOAPs, so there is no systematic variation in the optical absorption. Variations in $p_{0}$ with the number of pulses at a given wavelength originate from variations in the laser pulse energy for each pulse and noise in the data. Variations in $p_{0}$ between the two wavelengths are mainly due to the variation of the intrinsic absorption coefficient $\mu_{a}^{\left(b\right)}$ between these wavelengths. The pixel at (5.70, 4.06 mm) (indicated in blue in Fig. 8) is in a region of rsOAP accumulation. However, unlike the pixel at (7.34,−2.52  mm) (indicated in red), $p_{0}$ increases with the number of pulses at this location. This is because, for the considered sample structure, the laser fluence transmitted to this location is more heavily affected by the presence of proteins in other parts of the sample and increases with the number of pulses as $\mu_{a}^{\left(p\right)}$ decreases. Thus, for this pixel, the photoswitching is obscured, and in this case, pixel-level approaches^20^ and model-based approaches to unmixing^21^ are unlikely to provide accurate results. We note that of all the three locations, the reconstructed $p_{0}$ at (7.34,−2.52  mm) is the least affected by the noise in the data, even for high levels of noise. This is due to the stronger generated acoustic signal (compared with the location in the background) and the geometry of the transducer array, which causes it to have a higher detection sensitivity to OA signals originating from this region [compared with the pixel at (5.70, 4.06 mm)].

**Fig. 10:**
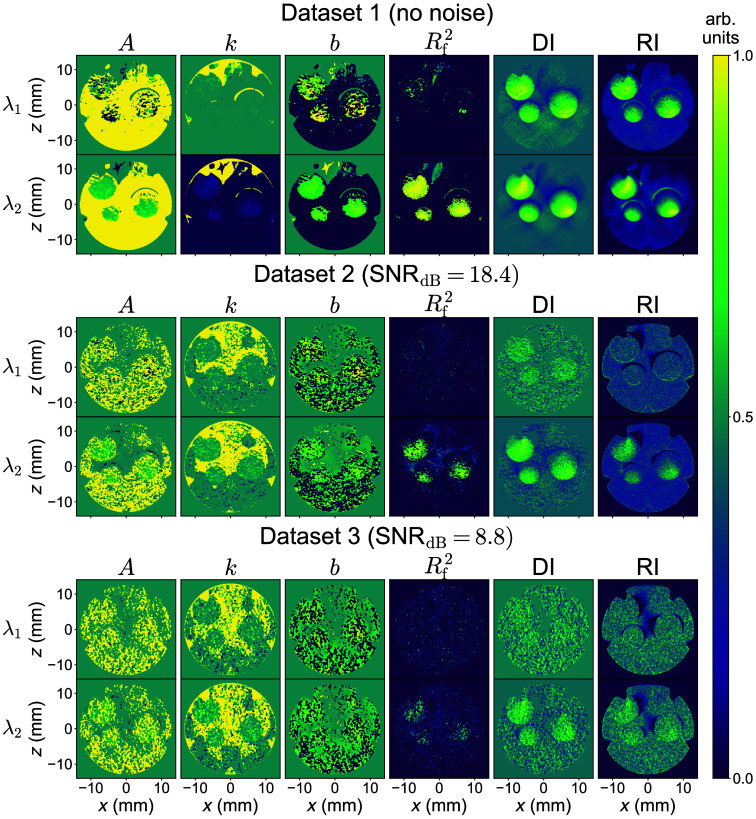
Normalized images of the 12 features extracted from the reconstructed images of the sample presented in Fig. 7, for dataset 1 with no noise in the data (top 2 rows), dataset 2 with signal-to-noise ratio ${\mathrm{SNR}}_{\mathrm{dB}}=18.4\;\mathrm{dB}$ (middle 2 rows), and dataset 3 with ${\mathrm{SNR}}_{\mathrm{dB}}=8.8\;\mathrm{dB}$ (bottom 2 rows). The features are: amplitude A, decay constant k, baseline b, goodness of fit $R_{f}^{2}$, differential imaging (DI), range image (RI) at wavelengths $\lambda_{1}=680$ and $\lambda_{2}=770\;\mathrm{nm}$. All images were individually min–max normalized to range from 0 to 1.

Normalized images of the 12 features extracted from the reconstructed images of the digital sample shown in Fig. 7 are presented in Fig. 10 for each dataset and wavelength. We note that the artifacts in the reconstructed images seen in Fig. 8 also affect the extracted features, especially DI and RI. In addition, as more noise is included, rsOAPs become more difficult to discern, but in all cases, rsOAPs are more visible in features extracted at 770 nm compared with features extracted at 680 nm. The exponential fit from Eq. (5) almost never achieved a good $R_{f}^{2}$ value (>0.7) when applied to images reconstructed from 680 nm laser pulses, even for noiseless data, because the optical contrast of the rsOAP at 680 nm does not change much when it photoswitches, compared with the change at 770 nm, which is much greater, as can be seen from Fig. 3.

### 3.4 Machine Learning

**Fig. 11:**
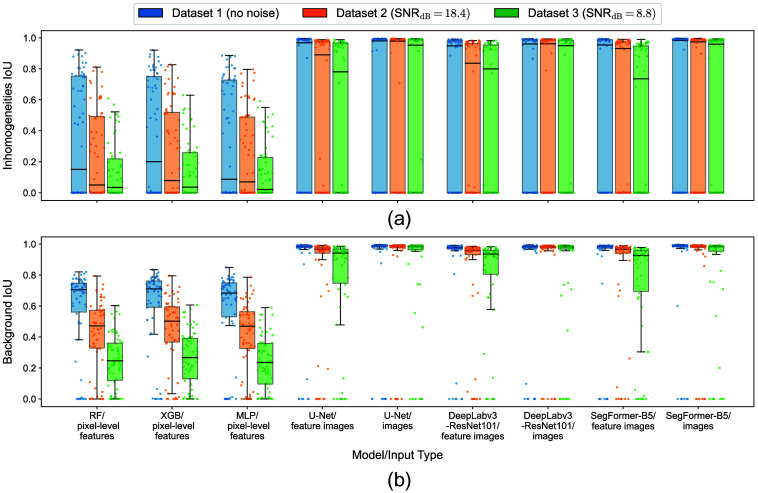
Box plots with jittered data points showing the intersection over union (IoU) of each model trained for binary semantic segmentation on the three datasets, for the inhomogeneities (a) and background (b) of each sample. Data points shown are the mean of each test sample over five repeats for each given model and dataset.

**Fig. 12:**
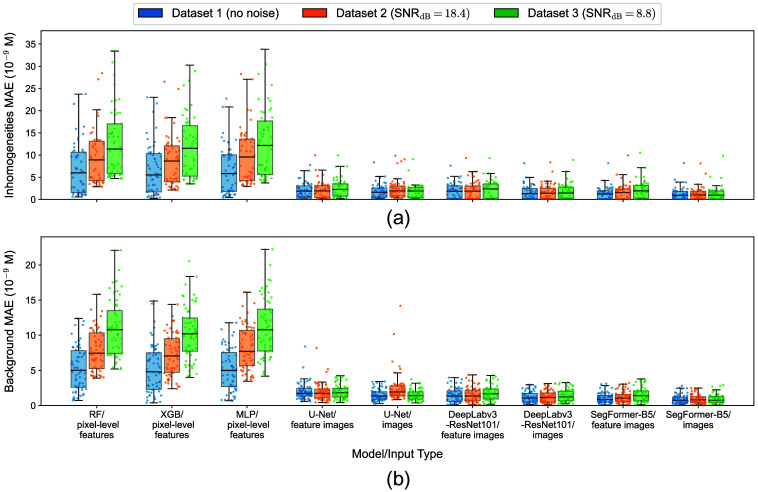
Box plots with jittered data points showing the mean absolute error (MAE) of each model trained for pixel-level prediction on the three datasets, for the inhomogeneities (a) and background (b) of each sample. Data points shown are the mean of each test sample over five repeats for each given model and dataset.

The test-set performance of each model is summarized in Figs. 11 and 12 and Tables 3–6. Each metric was computed per sample, for the inhomogeneities and background separately. Figure 11 shows the distribution of IoU on the test-set samples for the binary semantic segmentation task, and Fig. 12 shows the distribution of MAE on the test set samples for the pixel-level prediction task. Two outliers (both U-Net-ResNet101 trained for pixel-level prediction with feature images on dataset 1 with no noise) were excluded from the results because training failed to converge. Across both tasks and all datasets, U-Net-ResNet101, DeepLabV3-ResNet101, and SegFormer-B5 consistently outperformed RF, XGB, and MLP in all metrics for both the inhomogeneities and background regions. The performance of all models decreases as the noise in the data increases. All models consistently perform better on the background regions than on the inhomogeneities. The first quartile of the IoU for the inhomogeneities is around zero in Fig. 11 for all models and datasets, meaning the models misclassified the inhomogeneities in more than a quarter of the samples in every case.

**Table 3:** Test sensitivity and specificity on the inhomogeneities, of the models applied in the binary semantic segmentation of the reversibly switchable optoacoustic proteins (rsOAPs) for the three datasets. Values are shown as mean±95% confidence interval of the mean over all samples and five repeats.

Binary semantic segmentation test sensitivity and specificity (inhomogeneities)							
Model	Input	Dataset 1 (no noise)		Dataset 2 (${\mathrm{SNR}}_{\mathrm{dB}}=18.4$)		Dataset 3 (${\mathrm{SNR}}_{\mathrm{dB}}=8.8$)	
		Sensitivity	Specificity	Sensitivity	Specificity	Sensitivity	Specificity
RF	Pixel level	0.367	0.830	0.248	0.835	0.142	0.832
	Features	±0.002	±0.001	±0.001	±0.000	±0.003	±0.000
XGB	Pixel level	0.381	0.826	0.270	0.833	0.150	0.831
	Features	±0.000	±0.000	±0.001	±0.000	±0.000	±0.000
MLP	Pixel level	0.342	0.835	0.250	0.833	0.133	0.833
	Features	±0.008	±0.001	±0.016	±0.003	±0.022	±0.003
U-Net-	Feature	0.543	0.837	0.514	0.835	0.502	0.835
ResNet101	Images	±0.002	±0.000	±0.004	±0.000	±0.006	±0.001
U-Net-	Images	0.544	0.837	0.540	0.837	0.531	0.837
ResNet101		±0.003	±0.001	±0.007	±0.002	±0.006	±0.002
DeepLabV3-	Feature	0.541	0.836	0.508	0.834	0.503	0.833
ResNet101	Images	±0.004	±0.001	±0.002	±0.001	±0.006	±0.001
DeepLabV3-	Images	0.543	0.837	0.542	0.836	0.528	0.837
ResNet101		±0.002	±0.001	±0.005	±0.001	±0.002	±0.001
SegFormer-B5	Feature	0.543	0.837	0.511	0.835	0.494	0.832
	Images	±0.003	±0.000	±0.000	±0.001	±0.016	±0.003
SegFormer-B5	Images	0.545	0.838	0.544	0.837	0.527	0.837
		±0.002	±0.001	±0.004	±0.000	±0.002	±0.001

Tables 3 and 4 show the test sensitivity and specificity for binary semantic segmentation, for the inhomogeneities and background regions, respectively. The specificity (true negative rate) on the inhomogeneities was around 0.83 to 0.84 for all models and all datasets and was not affected by increasing the noise. By contrast, the sensitivity (true positive rate) of RF, XGB, and MLP, on the inhomogeneities was heavily affected by increasing noise, decreasing from around 0.34 to 0.37 with no noise to around 0.13 to 0.15 with high noise (${\mathrm{SNR}}_{\mathrm{dB}}=8.8$). The sensitivity of U-Net-ResNet101, DeepLabV3-ResNet101, and SegFormer-B5 on the inhomogeneities was much higher than that of RF, XGB, and MLP, and much less affected by increasing noise. It was also less affected when these models were trained on the reconstructed pressure images, instead of the extracted feature images. For example, the sensitivity of SegFormer-B5 on the inhomogeneities only decreased from 0.545 (no noise) to 0.527 (${\mathrm{SNR}}_{\mathrm{dB}}=8.8$) when it was trained on images, and from 0.543 to 0.494 when it was trained on feature images. The same trends are seen for the background specificity and sensitivity in Table 4, except both are much higher across all models and datasets for the background, with the background sensitivity being >0.98 in all cases, meaning there were almost zero false negatives in the background across all models.

**Table 4:** Test sensitivity and specificity on the background of the models applied in the binary semantic segmentation of the reversibly switchable optoacoustic proteins (rsOAPs) for the three datasets. Values are shown as mean±95% confidence interval of the mean over all samples and five repeats.

Binary semantic segmentation test sensitivity and specificity (background)							
Model	Input	Dataset 1 (no noise)		Dataset 2 (${\mathrm{SNR}}_{\mathrm{dB}}=18.4$)		Dataset 3 (${\mathrm{SNR}}_{\mathrm{dB}}=8.8$)	
		Sensitivity	Specificity	Sensitivity	Specificity	Sensitivity	Specificity
RF	Pixel level	0.619	0.990	0.445	0.993	0.279	0.986
	Features	±0.003	±0.001	±0.002	±0.000	±0.006	±0.001
XGB	Pixel level	0.643	0.989	0.475	0.991	0.300	0.985
	Features	±0.001	±0.000	±0.001	±0.000	±0.001	±0.000
MLP	Pixel level	0.586	0.997	0.448	0.990	0.262	0.989
	Features	±0.013	±0.001	±0.025	±0.004	±0.036	±0.006
U-Net-	Feature	0.879	0.999	0.836	0.998	0.790	0.996
ResNet-101	Images	±0.005	±0.000	±0.010	±0.001	±0.007	±0.002
U-Net-	Images	0.881	0.999	0.880	0.999	0.857	0.999
ResNet101		±0.002	±0.000	±0.003	±0.000	±0.014	±0.000
DeepLabV3-	Feature	0.875	0.998	0.830	0.997	0.798	0.996
ResNet-101	Images	±0.008	±0.000	±0.004	±0.001	±0.011	±0.000
DeepLabV3-	Images	0.881	0.999	0.879	0.999	0.858	0.998
ResNet101		±0.005	±0.000	±0.003	±0.001	±0.009	±0.000
SegFormer-B5	Feature	0.876	0.999	0.833	0.998	0.767	0.996
	Images	±0.003	±0.000	±0.005	±0.000	±0.015	±0.001
SegFormer-B5	Images	0.892	0.999	0.881	0.999	0.849	0.999
		±0.005	±0.001	±0.004	±0.001	±0.017	±0.000

Tables 5 and 6 show the test RMSE and MAE for pixel-level prediction, for the inhomogeneities and background regions, respectively. The trends seen in these tables are consistent with the binary semantic segmentation results; specifically, the RMSE and MAE of U-Net-ResNet101, DeepLabV3-ResNet101, and SegFormer-B5 are much lower than those of RF, XGB, and MLP and are much less affected by increasing noise.

**Table 5:** Test RMSE and MAE on the inhomogeneities of the models applied in the regression of the reversibly switchable optoacoustic proteins (rsOAPs) for the three datasets. Values are shown as mean±95% confidence interval of the mean over all samples and five repeats. Two outliers (both U-Net-ResNet101 trained with feature images on dataset 1 with no noise) were excluded from the results because training failed to converge.

Regression test RMSE and MAE (inhomogeneities)							
Model	Input	Dataset 1 (no noise)		Dataset 2 (${\mathrm{SNR}}_{\mathrm{dB}}=18.4$)		Dataset 3 (${\mathrm{SNR}}_{\mathrm{dB}}=8.8$)	
		RMSE	MAE	RMSE	MAE	RMSE	MAE
		($10^{-9}\;M$)	($10^{-9}\;M$)	($10^{-9}\;M$)	($10^{-9}\;M$)	($10^{-9}\;M$)	($10^{-9}\;M$)
RF	Pixel level	12.517	6.747	16.427	9.346	19.951	12.554
	Features	±0.013	±0.012	±0.014	±0.012	±0.012	±0.026
XGB	Pixel level	12.273	6.713	15.811	8.603	19.532	11.842
	Features	±0.026	±0.036	±0.013	±0.024	±0.021	±0.012
MLP	Pixel level	12.481	6.528	16.971	9.734	20.165	12.590
	Features	±0.143	±0.430	±0.124	±0.307	±0.190	±0.853
U-Net-	Feature	4.310	2.082	4.854	2.124	5.580	2.516
ResNet101	Images	±0.599	±0.405	±0.508	±0.365	±0.172	±0.100
U-Net-	Images	3.814	1.826	4.979	2.425	3.875	1.805
ResNet101		±0.396	±0.301	±2.220	±0.984	±0.584	±0.325
DeepLabV3-	Feature	4.221	1.870	4.630	2.054	5.013	2.270
ResNet101	Images	±0.109	±0.147	±0.582	±0.475	±0.579	±0.385
DeepLabV3-	Images	3.734	1.563	3.844	1.662	3.909	1.777
ResNet101		±0.218	±0.086	±0.306	±0.278	±0.346	±0.362
SegFormer-B5	Feature	3.361	1.342	4.122	1.670	5.014	2.149
	Images	±0.102	±0.112	±0.182	±0.074	±0.246	±0.101
SegFormer-B5	Images	2.962	1.201	3.015	1.216	3.168	1.302
		±0.159	±0.120	±0.143	±0.134	±0.081	±0.049

**Table 6:** Test RMSE and MAE on the background of the models applied in the regression of the reversibly switchable optoacoustic proteins (rsOAPs) for the three datasets. Values are shown as mean±95% confidence interval of the mean over all samples and five repeats. Two outliers (both U-Net-ResNet101 trained with feature images on dataset 1 with no noise) were excluded from the results because training failed to converge.

Regression test RMSE and MAE (background)							
Model	Input	Dataset 1 (no noise)		Dataset 2 (${\mathrm{SNR}}_{\mathrm{dB}}=18.4$)		Dataset 3 (${\mathrm{SNR}}_{\mathrm{dB}}=8.8$)	
		RMSE	MAE	RMSE	MAE	RMSE	MAE
		($10^{-9}\;M$)	($10^{-9}\;M$)	($10^{-9}\;M$)	($10^{-9}\;M$)	($10^{-9}\;M$)	($10^{-9}\;M$)
RF	Pixel level	11.675	5.358	15.121	7.836	18.889	10.959
	Features	±0.011	±0.013	±0.010	±0.015	±0.006	±0.019
XGB	Pixel level	11.318	5.263	14.530	7.181	18.458	10.353
	Features	±0.007	±0.020	±0.010	±0.021	±0.017	±0.009
MLP	Pixel level	11.552	5.039	15.607	8.209	19.118	11.122
	Features	±0.099	±0.468	±0.141	±0.364	±0.184	±0.976
U-Net-	Feature	4.776	1.999	5.300	1.946	5.622	1.978
ResNet101	Images	±0.651	±0.360	±1.352	±0.559	±0.162	±0.061
U-Net-	Images	3.980	1.528	6.051	2.474	3.972	1.493
ResNet101		±0.436	±0.270	±3.343	±1.224	±0.589	±0.249
DeepLabV3-	Feature	4.194	1.446	4.472	1.485	5.083	1.723
ResNet101	Images	±0.078	±0.141	±0.530	±0.349	±0.352	±0.250
DeepLabV3-	Images	3.734	1.182	3.758	1.233	3.864	1.368
ResNet101		±0.153	±0.081	±0.262	±0.219	±0.286	±0.305
SegFormer-B5	Feature	3.417	1.053	4.069	1.194	4.978	1.526
	Images	±0.037	±0.098	±0.162	±0.058	±0.152	±0.045
SegFormer-B5	Images	2.997	0.880	3.052	0.906	3.152	0.942
		±0.126	±0.085	±0.084	±0.079	±0.092	±0.027

**Fig. 13:**
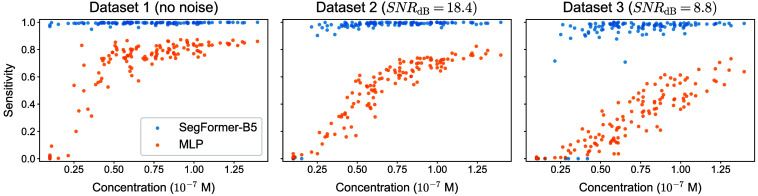
Sensitivity as a function of reversibly switchable optoacoustic protein (rsOAP) concentration, for the multilayer perceptron (orange), and SegFormer-B5 (blue), at each noise level. Each point corresponds to a given region containing rsOAPs, where the rsOAP concentration across each region is uniform as detailed in Sec. 2.2.

The sensitivity for the semantic segmentation task, as a function of concentration for the MLP, and SegFormer-B5, at each noise level, is presented in Fig. 13. The MLP and SegFormer-B5 were selected for Fig. 13 to represent the two broad categories of approaches considered in this study: those that do not exploit spatial context (the tree ensembles and the MLP) and those that do (the CNNs and the transformer). The maximum sensitivity for the MLP is ∼0.85 and gradually decreases with concentration, dropping below 0.5 at approximately $0.4\times 10^{-7}$, $0.5\times 10^{-7}$, and $0.6\times 10^{-7}\;M$, for dataset 1 (no noise), dataset 2 (${\mathrm{SNR}}_{\mathrm{dB}}=18.4$), and dataset 3 (${\mathrm{SNR}}_{\mathrm{dB}}=8.8$), respectively. By contrast, SegFormer-B5 usually achieves ∼1.0 or ∼0.0 sensitivity on a given region. SegFormer-B5 achieved ∼1.0 sensitivity for dataset 1 (no noise), ∼0.0 sensitivity below $0.2\times 10^{-7}\;M$ for dataset 2 (${\mathrm{SNR}}_{\mathrm{dB}}=18.4$), and ∼0.0 sensitivity for some inhomogeneities below about 0.3 to $0.4\times 10^{-7}\;M$ for dataset 3 (${\mathrm{SNR}}_{\mathrm{dB}}=8.8$).

**Fig. 14:**
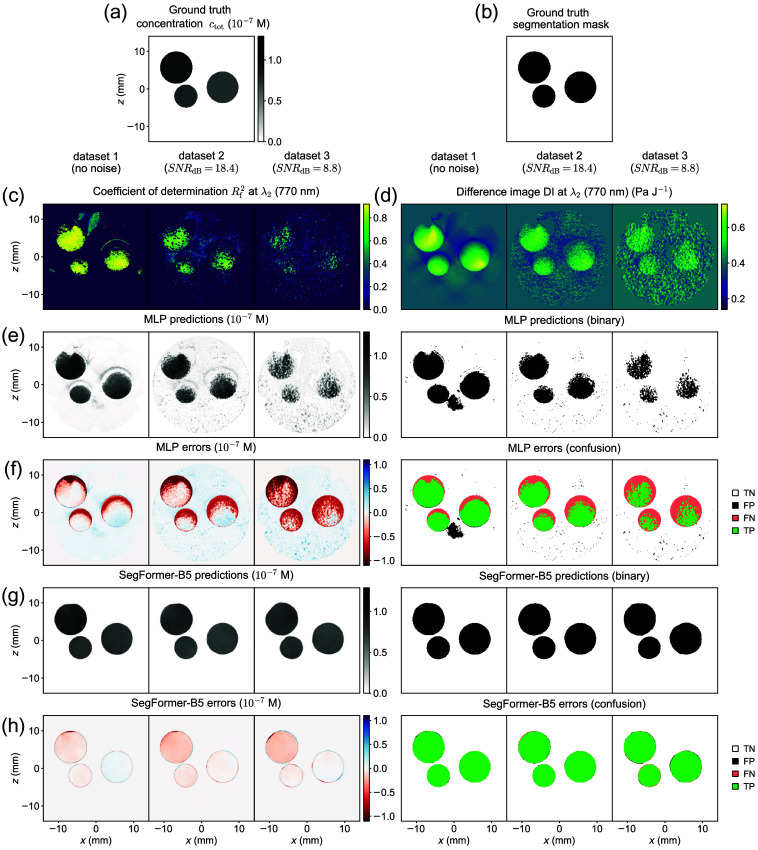
Ground truth reversibly switchable optoacoustic protein (rsOAPs) concentration (a) and ground-truth segmentation mask (b), fit coefficient of determination $R_{f}^{2}$ (c) and difference image DI (d). Predictions and errors for multilayer perceptron (MLP) [(e) and (f), respectively] and SegFormer-B5 [(g) and (h), respectively] on pixel-level prediction (left side), and binary semantic segmentation (right side). Semantic segmentation errors are labeled as white = true negative (TN), black = false positive (FP), pink = false negative (FN), and green = true positive (TP).

Figure 14 presents the predictions and errors on both tasks, using the MLP and SegFormer-B5, at each noise level when the models were trained using the extracted feature images. More of these comparisons are shown in Sec. 6.2 in the Appendix. The MLP performs well when the extracted features are informative, but the features are negatively affected as more noise is added. In particular, for dataset 3, the exponential fit [Eq. (5)] almost always fails to converge, and the MLP performs poorly. Even with no measurement noise, artifacts in the reconstructed images of the acoustic pressure can affect the features. Most notable are artifacts due to the limited angular coverage of the MSOT’s transducer array, which appear in the upper half of the samples and inhomogeneities in the images. In these regions, the MLP typically fails its predictions, whereas the predictions of SegFormer-B5 remain highly accurate. The errors in SegFormer-B5’s predictions at the interface between inhomogeneities and background are attributed to blurring of boundaries between tissue types due to the point spread function of the imaging system.

## 4 Discussion

### 4.1 Forward Model Validation

The simulations in this study incorporated the key physical processes associated with OA imaging of rsOAPs, specifically light transport, photoswitching, and acoustic wave propagation. Reconstructed images from simulated and experimental data exhibit similar characteristics (Fig. 5). The decrease in the reconstructed OA signal toward the top of both the simulated and experimental images is attributed to the limited angular coverage of the MSOT’s transducer array. In the simulated images, the spatial artifacts that trace the paths of the illuminating light through the sample and their intersection are more pronounced. This may be because, in physical experiments, light emitted by the MSOT’s light source is more diffuse, and the optical scattering coefficient of the experimental sample may be larger than that of the digital sample. Because of this, and other differences in the optical properties of the simulation and experimental samples, quantitative agreement between the decay constants k when fitting Eq. (5) was not obtained. However, in both the simulated and experimental data, the initial pressure from the rsOAPs decays exponentially to a baseline value during successive imaging, which is in agreement with Eq. (2) and is consistent with previous studies.^3^^,^^6^ In both cases, a high coefficient of determination was obtained by fitting the reconstructed OA signals extracted from the ROI to Eq. (5).

In this study, it was assumed that photoswitching occurs instantaneously at the end of each illumination. Other phytochromes that have been studied switch between the two metastable forms (Pr and Pfr) by transitioning through multiple intermediate isomers, which decay via vibrational relaxation. The first intermediates can form ∼10 to 100 ps after photoexcitation, and subsequent intermediates often take longer to decay.^33^ The intermediate isomers of full-length DrBphP were found to have time constants of 80  μs, 2.6, and 27 ms.^60^ This means that changes in the concentration of Pr, Pfr, any intermediates, and the corresponding absorption spectra can occur continuously and on a similar timescale to the laser pulse duration (∼10  ns^30^). In such situations, as light absorption decreases with photoswitching, the optical simulation underestimates the photon fluence. One way of mitigating this would be to split each laser pulse into several time-steps, and run MCX before updating $c_{\mathrm{Pr}}$ and $c_{\mathrm{Pfr}}$ for each time step. This was not performed in this study due to computational constraints, as the optical simulation time scales linearly with the number of time-steps. However, by assuming that ReBphP-PCM switches between Pr and Pfr or vice versa instantly upon photoexcitation, it was possible to replicate experimental behavior in which the contrast decays exponentially with the number of laser pulses (Figs. 5 and 6).

As a trade-off between model complexity and realism, two simplifying assumptions were adopted when generating the synthetic data used in this study. First, the additional absorption associated with the tumor-like inhomogeneity was assumed to be additive, which does not necessarily hold in solid tumors, where tumor cells can occupy a substantial fraction of the tissue volume. Second, the background optical coefficients at the two wavelengths were sampled independently from identical distributions, whereas real biological tissues exhibit systematic spectral correlations in their optical properties. These simplifications do not affect the conclusion of the current study, as the analysis targets the time-varying photoswitching contrast rather than the static background, but addressing them would improve the realism of synthetic datasets for training on experimental data, and avoid domain shift. Building on the model presented here, future studies based on synthetic data may also consider acoustic attenuation, media with inhomogeneous acoustic properties, greater variety in the samples (for instance, samples including inhomogeneities of various shapes), system-dependent image noise modeling, and forward model calibration^38^ with real data when available. The use of additive, uncorrelated Gaussian noise provides a controlled way to vary the signal-to-noise ratio and assess robustness. However, real optoacoustic measurements may exhibit non-Gaussian, multiplicative, or correlated noise characteristics.

### 4.2 Machine Learning

The ML experiments in this study demonstrate that, with accurately labeled ground truth, supervised ML techniques can enable the semantic segmentation and quantification of rsOAPs from synthetic OA imaging data, even with high levels of measurement noise (Figs. 11 and 12 and Tables 3–6). Unlike existing model-based unmixing approaches,^21^ this study demonstrated that CNNs and a transformer neural network are capable of predicting rsOAP concentrations without prior knowledge of the intrinsic optical properties of the sample, instead learning directly from reconstructed temporal image sequences (or feature images derived from them). In line with prior work,^7^ classical ML models based on pixel-level engineered temporal features (RF, XGB, and MLP) provided useful baselines. RF, XGB, and MLP all achieved similar performance (Figs. 11 and 12) and were limited by their lack of spatial modeling. By contrast, U-Net-ResNet101, DeepLabV3-ResNet101, and SegFormer-B5 consistently achieved higher IoU/sensitivity in the binary semantic segmentation task and lower RMSE/MAE in the regression task across all noise levels (Figs. 11 and 12 and Tables 3–6). This is attributed to their ability to combine spatial and temporal information, which can improve robustness to image reconstruction artifacts (e.g., those seen in Fig. 8), high-noise levels, spectral coloring, and shadowing.

Despite hyperparameter optimization only being performed for RF, XGB, and MLP, these models were still vastly outperformed by the CNNs and SegFormer-B5, which were trained in their default configurations. It was not computationally feasible to perform hyperparameter optimization for U-Net-ResNet101, DeepLabV3-ResNet101, and SegFormer-B5 in this study, which remains the subject of future studies and would enable a more systematic comparison of different model architectures. The evaluated models differ in several aspects, including model capacity, inductive biases (e.g., spatial modeling), use of pretraining, and degree of hyperparameter optimization. As a result, their relative performance cannot be attributed to a single factor. Instead, this study assesses the performance of different classes of models when trained on identical synthetic datasets under varying noise conditions.

The U-Net-ResNet101, DeepLabV3-ResNet101, and SegFormer-B5 performed better when trained using the temporal image sequences, as opposed to the feature images. This improvement was greater when more noise was included in the data, with mean RMSE on the inhomogeneities decreasing by 11.7% with dataset 1 (no noise), 13.8% with dataset 2 (${\mathrm{SNR}}_{\mathrm{dB}}=18.4$), and by 29.8% with dataset 3 (${\mathrm{SNR}}_{\mathrm{dB}}=8.8$), between the three models when predicting protein concentrations. This shows that feature extraction is not necessary to achieve strong performance with these models; instead, they derive task-relevant features directly from the input data.^61^ These learned features are more difficult to interpret than engineered features but encapsulate more information relevant to the segmentation and quantification tasks. Hybrid approaches that combine learned representations with more interpretable engineered features^62^ may therefore be a promising direction.

Using the data in this study, the performance of the transformer (SegFormer-B5) was similar to the CNNs (U-Net-ResNet101 and DeepLabV3-ResNet101) for the semantic segmentation task. Specifically, across all three datasets, SegFormer-B5 achieved a mean IoU score of 0.535 for the inhomogeneities and 0.864 for the background, whereas the CNNs achieved mean IoU scores of 0.533 for the inhomogeneities and 0.863 for the background. However, SegFormer-B5 performed better than the CNNs on the pixel-level prediction task. Specifically, SegFormer-B5 achieved a mean MAE of $1.240\times 10^{-9}\;M$ for the inhomogeneities and $0.910\times 10^{-9}\;M$ for the background when predicting concentration, whereas the CNNs achieved mean MAEs of $1.843\times 10^{-9}\;M$ for the inhomogeneities and $1.546\times 10^{-9}\;M$ for the background.

Figure 13 shows that model sensitivity is concentration-dependent: the MLP exhibits a gradual fall-off in sensitivity as the rsOAP concentration decreases, with the concentration at which performance degrades shifting to higher values as noise increases. By contrast, SegFormer-B5 displays a sharper transition between near-perfect detection and near-complete failure for a given inhomogeneity.

While exhibiting excellent performance so far, the CNN and transformer models are computationally expensive and require large quantities of data to train compared with the RF, MLP, and XGB; the latter models may perform better than the CNNs and transformers when training data is extremely limited. The high costs and challenges of obtaining and labeling large quantities of experimental data means that moving forward, it is important to explore approaches that require less training data to achieve good performance and generalization on unseen data. Furthermore, the CNN and transformer model’s reliance on spatial information could cause their performance to degrade if there are changes in the distribution of spatial features in the input data compared with the training data. This problem is known as domain shift and is less likely to affect approaches which only use temporal information such as RF, XGB, or MLP, which, consequently, might prove more robust if the spatial structure of the test samples differs greatly from the training samples. Domain transfer techniques, such as fine-tuning^63^ or domain adaptation,^64^ can be used to mitigate the impact of domain shifts. Pre-training a neural network on synthetic data, such as the datasets created in this work, before fine-tuning it on clinical or preclinical data will likely result in faster convergence and better generalization if there is insufficient real data to fully train the models.

This study trained and evaluated models separately for binary semantic segmentation and pixel-level prediction. Future work may make use of both synthetic data and experimental data (with reference data from histology), perform a systematic comparison between masks obtained by thresholding concentration predictions and masks produced by dedicated semantic segmentation models, examine how segmentation and quantification performance depend on inhomogeneity size and position relative to the detector array, and adopt boundary-based metrics such as the 95th-percentile Hausdorff distance where boundary fidelity is important for downstream use.

## 5 Conclusion

In summary, this study is the first to demonstrate the feasibility of deep learning for the optoacoustic imaging of reversibly switchable optoacoustic proteins. A forward model for data generation was developed and used to generate a synthetic dataset for machine learning experiments. This forward model provides a resource for testing and refining methods for semantic segmentation and quantification of reversibly switchable optoacoustic proteins. It was shown that CNNs and transformers, by analyzing both spatial and temporal information, can greatly outperform machine learning methods that use only engineered temporal features, an advantage that would be maintained so long as the spatial information remains consistent between training and test data. These results pave the way for further developments, including evaluating the machine learning models using experimental datasets, and potentially applying deep learning for quantitative imaging of CAR T-cells in immunotherapy.

## 6 Appendix: Photoswitching Model Derivation and Additional Test Examples

### 6.1 Photoswitching Model

The following is a derivation of Eq. (2). The time between successive images taken with the MSOT is 100 ms, and the duration of each pulse is ∼10  ns. In this time-frame the effects of photobleaching, photofatigue, and dark decay^6^^,^^33^ are negligible, and photoswitching is a one-photon process.^3^^,^^5^ In this case, the rate of change in the concentration of proteins in the Pr state when irradiated by monochromatic light obeys the equation

$$\frac{dc_{\mathrm{Pr}}(r,\lambda ,t)}{dt}=\frac{\lambda \phi (r,\lambda ,t)}{hcN_{A}}[\eta_{\mathrm{Pfr}}(\lambda )\epsilon_{\mathrm{Pfr}}(\lambda )c_{\mathrm{Pfr}}(r,t)-\eta_{\mathrm{Pr}}(\lambda )\epsilon_{\mathrm{Pr}}(\lambda )c_{\mathrm{Pr}}(r,t)].$$ (13)

Here, h is Planck’s constant, c is the speed of light, $N_{\text{A}}$ is Avogadro’s constant, φ is the illumination flux, $\eta_{\mathrm{Pr}}$ and $\eta_{\mathrm{Pfr}}$ are the photoswitching efficiencies for the Pr to Pfr and the Pfr to Pr transitions, respectively, and $\epsilon_{\mathrm{Pr}}$ and $\epsilon_{\mathrm{Pfr}}$ are the molar absorption coefficients corresponding to the Pr and Pfr states, respectively. Using $c_{\mathrm{tot}}=c_{\mathrm{Pr}}+c_{\mathrm{Pfr}}$, Eq. (13) can be expressed as

$$\frac{dc_{\mathrm{Pr}}(r,\lambda ,t)}{dt}=\frac{\lambda \phi (r,\lambda ,t)}{hcN_{A}}[\eta_{\mathrm{Pfr}}(\lambda )\epsilon_{\mathrm{Pfr}}(\lambda )c_{\mathrm{tot}}(r)-c_{\mathrm{Pr}}(r,\lambda ,t)(\eta_{\mathrm{Pfr}}(\lambda )\epsilon_{\mathrm{Pfr}}(\lambda )+\eta_{\mathrm{Pr}}(\lambda )\epsilon_{\mathrm{Pr}}(\lambda ))].$$ (14)

If the sample is illuminated by laser pulse i at $t_{i}=(i-1)\Delta t$, where Δt is the time between laser pulses, using $c_{\mathrm{Pr}}(t_{i})$, we can solve Eq. (14) for $c_{\mathrm{Pr}}(t_{i}+1)$,

$$c_{\mathrm{Pr}}(r,\lambda ,t_{i+1})=\alpha (r,\lambda )+[c_{\mathrm{Pr}}(r,\lambda ,t_{i})-\alpha (r,\lambda )]\exp[-\beta (\lambda )\Phi_{i}(r,\lambda )],$$ (15)

where,

$$\alpha (r,\lambda )=\frac{\eta_{\mathrm{Pfr}}(\lambda )\epsilon_{\mathrm{Pfr}}(\lambda )c_{\mathrm{tot}}(r)}{\eta_{\mathrm{Pfr}}(\lambda )\epsilon_{\mathrm{Pfr}}(\lambda )+\eta_{\mathrm{Pr}}(\lambda )\epsilon_{\mathrm{Pr}}(\lambda )},$$ (16)

$$\beta (\lambda )=\frac{\lambda }{hcN_{A}}(\eta_{\mathrm{Pfr}}(\lambda )\epsilon_{\mathrm{Pfr}}(\lambda )+\eta_{\mathrm{Pr}}(\lambda )\epsilon_{\mathrm{Pr}}(\lambda )),$$ (17)

and,

$$\Phi_{i}(r,\lambda )=\int_{t_{i}}^{t_{i+1}}\phi (r,\lambda ,t)dt.$$ (18)

**Fig. 15:**
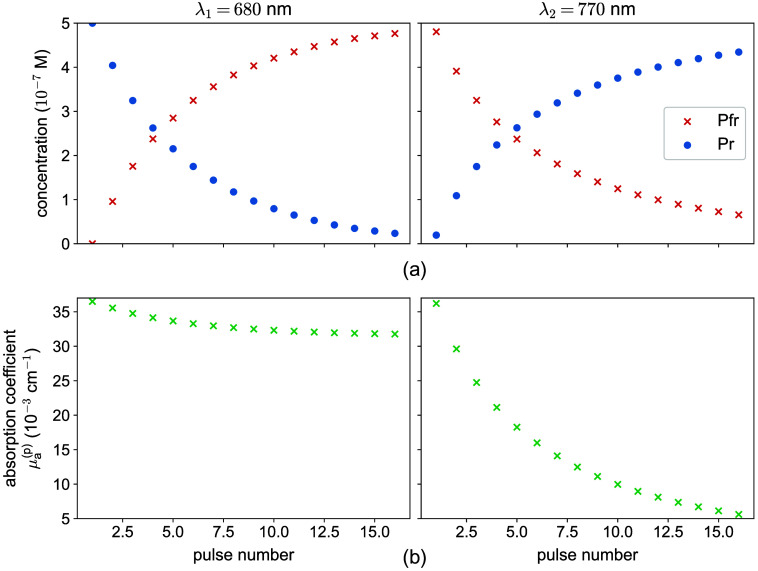
(a) The concentrations of far-red-absorbing (Pfr) and the red-absorbing (Pr) forms of the reversibly switchable optoacoustic protein (rsOAP) as a function of laser pulse number for the two excitation wavelengths, for a voxel in Fig. 5. (b) Optical absorption coefficient of the rsOAPs ($\mu_{a}^{\left(p\right)}$) at 680 nm (left) and 770 nm (right). First the rsOAP is imaged using 16 laser pulses with 680 nm light (left), followed by 16 pulses with 770 nm light.

The concentration of proteins in the Pfr state can then be obtained as $c_{\mathrm{Pfr}}=c_{\mathrm{tot}}-c_{\mathrm{Pr}}$. A result of Eq. (15) is that $c_{\mathrm{Pr}}$ and $c_{\mathrm{Pfr}}$ at time $t_{i+1}$ depend on the fluence up to time $t_{i+1}$, not the duration of the pulses. In this study, it was assumed that $\eta_{\mathrm{Pr}}(\lambda_{2})=\eta_{\mathrm{Pfr}}(\lambda_{1})=0$, whereas $\eta_{\mathrm{Pr}}(\lambda_{1})=0.004$ and $\eta_{\mathrm{Pfr}}(\lambda_{2})=0.002$. Figure 15 shows how $c_{\mathrm{Pr}}$, $c_{\mathrm{Pfr}}$, and the optical absorption coefficient of the rsOAPs evolve over sequential imaging according to Eq. (15).

### 6.2 Best and Worst Test Examples

**Fig. 16:**
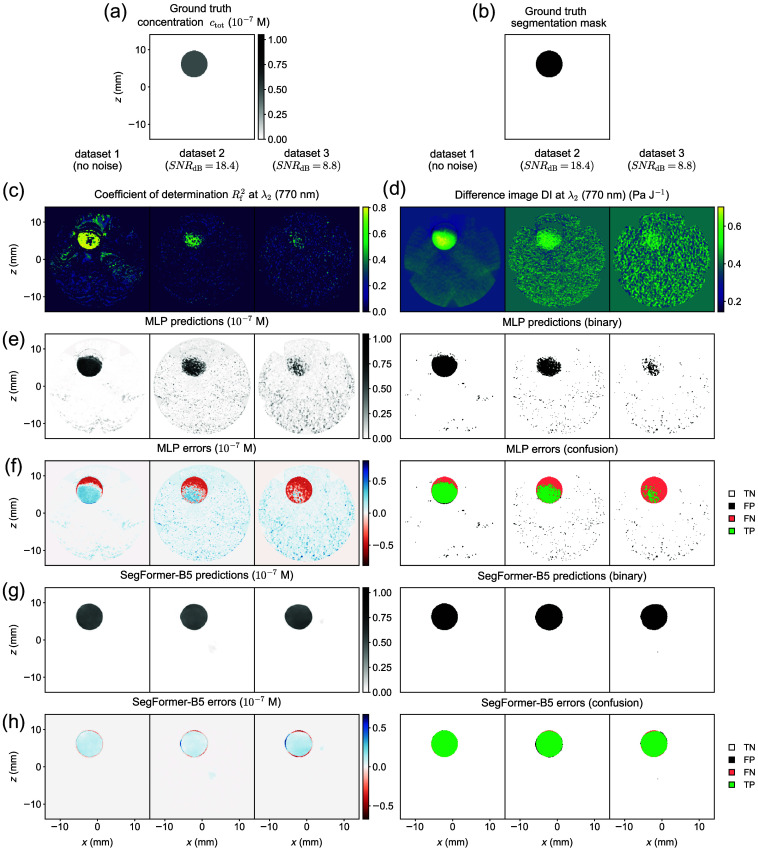
Predictions on best-case test-set sample. Ground truth reversibly switchable optoacoustic protein (rsOAPs) concentration (a) and ground-truth segmentation mask (b), fit coefficient of determination $R_{f}^{2}$ (c) and difference image DI (d). Predictions and errors for multilayer perceptron (MLP) [(e) and (f), respectively] and SegFormer-B5 [(g) and (h), respectively] on pixel-level prediction (left side), and binary semantic segmentation (right side). Semantic segmentation errors are labeled as white = true negative (TN), black = false positive (FP), pink = false negative (FN), and green = true positive (TP).

**Fig. 17:**
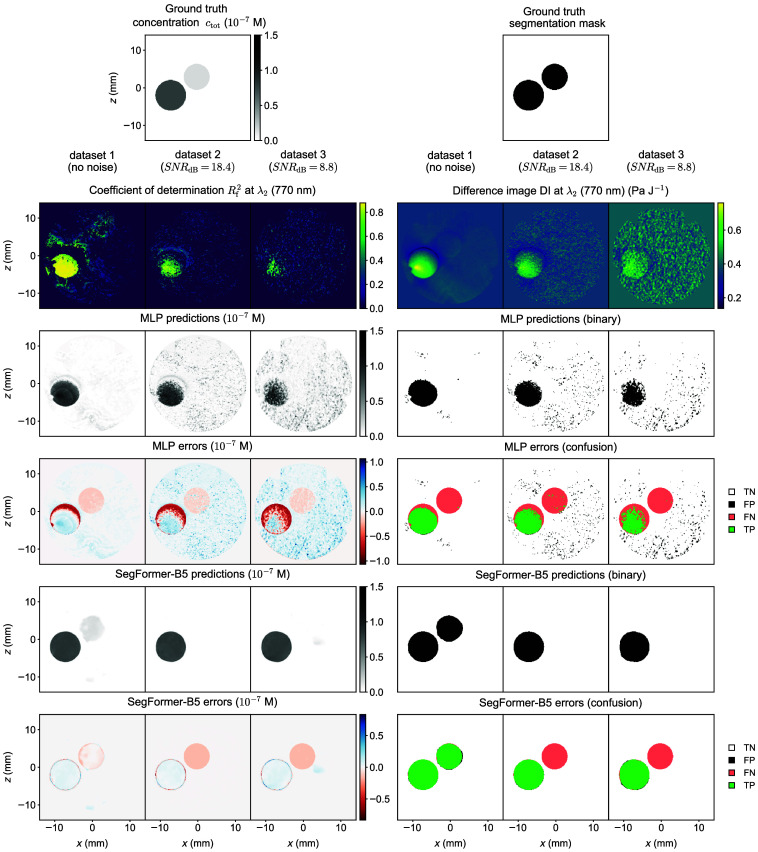
Same as Fig. 16 but for the worst test-set sample.

Predictions and errors on two test-set samples for the MLP and SegFormer-B5, for both semantic segmentation and pixel-level prediction, for all three noise levels, are presented in Figs. 16 and 17, with Fig. 16 showing the best and Fig. 17 showing the worst. The worst was defined as the sample that placed in the bottom three the most times across all metrics (background and inhomogeneities), noise levels, both tasks, for MLP and SegFormer-B5. The best was defined in the same way except by placing in the top 3 the most times, and samples containing no proteins were excluded from the comparison.

## Data Availability

The code used in the creation of the synthetic dataset is available at https://github.com/bvale1/python_BphP_MSOT_sim. The code used for data analysis and machine learning experiments is available at https://github.com/bvale1/BphPSEG. Data will be made available upon request.
